# Molecular Basis of Virulence in *Staphylococcus aureus* Mastitis

**DOI:** 10.1371/journal.pone.0027354

**Published:** 2011-11-11

**Authors:** Caroline Le Maréchal, Nubia Seyffert, Julien Jardin, David Hernandez, Gwenaël Jan, Lucie Rault, Vasco Azevedo, Patrice François, Jacques Schrenzel, Maarten van de Guchte, Sergine Even, Nadia Berkova, Richard Thiéry, J. Ross Fitzgerald, Eric Vautor, Yves Le Loir

**Affiliations:** 1 INRA, UMR1253, Science et Technologie du Lait et de l'Œuf, Rennes, France; 2 AGROCAMPUS OUEST, UMR1253, Science et Technologie du Lait et de l'Œuf, Rennes, France; 3 ANSES, Laboratoire de Sophia-Antipolis, Unité pathologie des ruminants, Sophia-Antipolis, France; 4 Universidade Federal de Minas Gerais (UFMG), Instituto de Ciências Biológicas (ICB), Departamento de Biologia Geral, Belo Horizonte, Minas Gerais, Brazil; 5 Genomic Research Laboratory, Service of Infectious Diseases, University of Geneva Hospitals HUG, Geneva, Switzerland; 6 INRA, UMR1319, MICALIS, Jouy en Josas, France; 7 The Roslin Institute and Centre for Infectious Diseases, Royal Dick School of Veterinary Studies, University of Edinburgh, Edinburgh, Scotland, United Kingdom; University of Liverpool, United Kingdom

## Abstract

**Background:**

*S. aureus* is one of the main pathogens involved in ruminant mastitis worldwide. The severity of staphylococcal infection is highly variable, ranging from subclinical to gangrenous mastitis. This work represents an in-depth characterization of *S. aureus* mastitis isolates to identify bacterial factors involved in severity of mastitis infection.

**Methodology/Principal Findings:**

We employed genomic, transcriptomic and proteomic approaches to comprehensively compare two clonally related *S. aureus* strains that reproducibly induce severe (strain O11) and milder (strain O46) mastitis in ewes. Variation in the content of mobile genetic elements, iron acquisition and metabolism, transcriptional regulation and exoprotein production was observed. In particular, O11 produced relatively high levels of exoproteins, including toxins and proteases known to be important in virulence. A characteristic we observed in other *S. aureus* strains isolated from clinical mastitis cases.

**Conclusions/Significance:**

Our data are consistent with a dose-dependant role of some staphylococcal factors in the hypervirulence of strains isolated from severe mastitis. Mobile genetic elements, transcriptional regulators, exoproteins and iron acquisition pathways constitute good targets for further research to define the underlying mechanisms of mastitis severity.

## Introduction

Mastitis is an inflammation of the mammary gland with local and or general symptoms that occasionally result in a systemic infection. This disease has a profound impact on animal welfare and milk quality [1] leading to great economical losses in milk production [2]. *Staphylococcus aureus* is a major cause of mastitis in ruminants worldwide which is often difficult to cure and is prone to resurgence. Beside mastitis, *S. aureus* is involved in a wide range of infections. In several infection types (e.g. pneumonia, osteomyelitis, skin infections), extremely severe cases associated with hypervirulent strains have been reported [3]–[6]. The existence of hypervirulent strains emphasizes the need to define the strain characteristics involved in the increased severity so as to better monitor their dissemination and find relevant therapeutic targets to reduce severity. It has been reported that severity can be linked to the production of a single virulence factor that enhances the virulence of producing strains. For example, Panton-Valentine leukocidin, a bi-component pore-forming toxin, is particularly prevalent in severe infections [4] and has been proposed as a hypervirulent determinant [7], due to its involvement in leukocyte destruction and tissue necrosis [8], [9]. Furthermore, staphylococcal superantigens or alpha-toxin function in a dose-dependant manner, resulting in more severe infections caused by highly-expressing strains [10]–[13]. Severity of mastitis caused by *Escherichia coli* was shown to be mainly determined by host factors and not by the strains features [14]. In contrast, in *S. aureus* mastitis, inter-strain variations exist in terms of virulence potential [15]. Alpha-toxin and LukM-F' have been reported to be highly produced during gangrenous *S. aureus* mastitis [13], [16]–[19]. However, global studies which examine the expression of all proteins have not been carried out, and to date no gene has been identified as being a severity marker [20]–[22]. A better understanding of the pathogenicity of *S. aureus* is critical to develop more efficient and satisfactory therapy to overcome mastitis.

*S. aureus* strains O11 and 046 were isolated from gangrenous mastitis and subclinical mastitis of ewes, respectively. These strains were shown to reproducibly induce severe (O11) or mild (O46) mastitis in experimental infections [15]. In the current study, they were comprehensively analyzed by a comparative genomic, transcriptomic and proteomic approach to identify staphylococcal factors that can be linked to mastitis severity in order to define strain characteristics associated with hypervirulence in mastitis.

## Results

### Genome analysis reveals minor differences between O11 and O46

**Figure 1:**
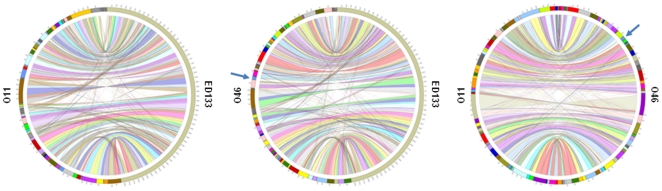
Graphical mapping of the genomes of *S. aureus* O11 and O46 and the recently released *S. aureus* ED133 genome. Left panel: *S. aureus* O11 (left side) and *S. aureus* ED133 (right side), middle panel: *S. aureus* O46 (left side) and ED133 (right side) and right panel: O11 (left side) and O46 (right side) genomes. Homologuous sequences between strains are linked by colored ribbons. Sequences are ordered in a way to minimize ribbons crossing. Arrows indicate the contig containing the additional phage in O46 (see text for details).

In order to investigate the genetic bases for the high virulence of strain O11 in ewe mastitis, we determined and compared the genome sequences of strains O11 and O46 [23]. The great majority of the genes were found in both strains except for an additional serogroup B prophage (42 CDS) in O46 genome (Figure 1). O11 and O46 share high similarity with the recently sequenced ED133 genome [24] (Figure 1), a *S. aureus* strain isolated from ovine mastitis. Yet, ED133 belongs to the clonal complex CC133 (MLST) whereas O11 and O46 clustered in the same lineage as bovine strains found in CC130 [25]. In a study by Guinane et al, comparative genome analysis of ED133 in addition to other ruminant and human strains revealed molecular evidence for host-adaptation and several novel mobile genetic elements (MGE) encoding virulence proteins with attenuated or enhanced activity in ruminants [19]. In the current study, we found that most of the genes present in ED133 genome are present in O11 or O46 genomes (Figure 1). For example, both O11 and O46 carry the newly described phages related to the φSaov1 and φSaov3 phages from ED133 but do not contain φSaov2, reportedly unique to ED133, or SaPIov1, carrying an ovine allelic variant of *sec* (encoding staphylococcal enterotoxin type C). Nevertheless *scn* (staphylococcal complement inhibitor), *vwb* (von Willebrand factor-binding protein) and SAOV_2050 (hypothetical protein) carried by SaPIov2 pathogenicity island are identified in O11 and O46 sequences. In contrast to ED133, putative virulence factors *edin-B* and a homolog of *etd* carried by a putative pathogenicity island are present in both O11 and O46 [26].

Although O11 and O46 are clonally related as demonstrated by *spa* typing, PFGE analysis [27] and genome content, they contain Single Nucleotide Polymorphims (SNP) (around 1600 synonymous SNP and 1250 non synonymous SNP detected). SNP mediated diversification of genes encoding cell-wall associated proteins was previously observed [24], [28]. Here, comparison of O11 and O46 showed that the SNPs were evenly distributed around the genome and did not correlate with protein location or function.

O11 and O46 comparison also revealed 103 truncated genes (listed in supplemental data, Table S1) present in one strain or the other, corresponding to point mutations or indels causing a frameshift or leading to a premature stop codon. Among these 103 truncated genes, 37% are involved in cellular machinery, notably in gene regulation (8.7%), iron metabolism (3%), virulence (11%), and proteins of unknown function (36%). Truncated genes that may play a role in phenotype differences observed between O11 and O46 have been identified. For instance, 2 genes encoding enzymes involved in restriction/modification systems are found intact in O46 (046_2610 similar to type III restriction protein [29] and 046_0485 similar to HsdR type I restriction endonuclease [30] whereas they are truncated in O11 (Table S1). Transformation tests (electroporation with pMAD plasmid DNA directly extracted from *E.coli* DH5) on O11 and O46 revealed that only O11 is transformable with transformation efficiency comparable to that of *S. aureus* RN4220, bearing the same mutations [30]. Plasmidic DNA extracted from O11 transformants was successfully introduced into *S. aureus* MW2 and, to a lesser extend, into O46, suggesting that additional feature(s) impairs O46 transformability (see supplemental data, Table S2). Similarly, *icaC* is truncated in O11, and this correlates with a lower capacity for biofilm formation in O11 when compared to O46 (biofilm formation tested as described in [31]; see supplemental data, figure S1). Some of these differences have direct consequences on transcription as revealed by transcriptomic differences (18% of the truncated genes appeared underexpressed in O11 or O46 (Table S1)).

### Comparison of O11 and O46 transcriptome during growth in mastitis-like conditions reveals major differences

**Figure 2:**
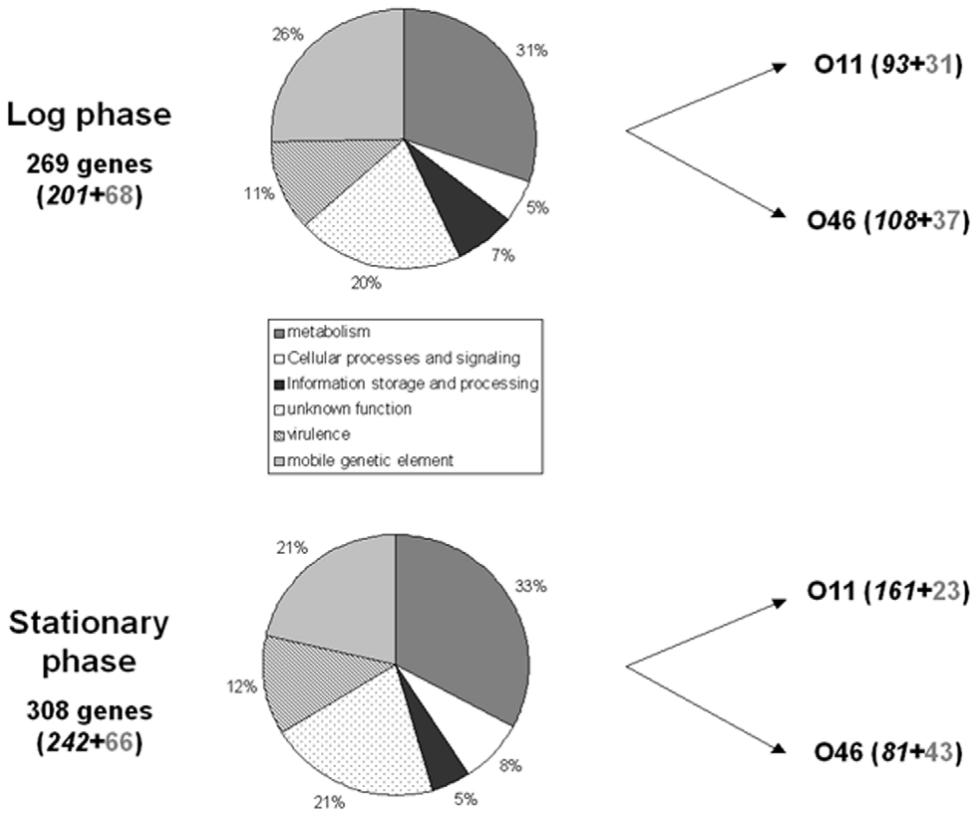
Transcriptomic comparison of *S. aureus* O11 and *S. aureus* O46 after growth in deferoxamine-RPMI during log and stationary phase. Genes differentially expressed were categorized by functional annotation. Genes overexpressed by each strain are indicated on the right side of the figure. Right side, numbers of genes differentially expressed belonging to the core genome (italic black) and mobile genetic elements (grey) are indicated.

Total RNA samples were prepared from O11 and O46 strains grown in deferoxamine-RPMI under anaerobic conditions to simulate the *in vivo* context [32]. Cells were harvested in exponential and stationary phase, and gene expression profiles determined. Fold changes in Table S3, S4, and S5 indicate the gene expression ratio between O11 and O46. Only ratios higher than 2 (overexpression in O11) and lower than 0.5 (overexpression in O46) were considered. Microarray analyses showed that 269 genes and 308 genes (Table S4 and S5) were differentially expressed between O11 and O46 during log and stationary phases respectively (Figure 2). The two strains had significantly different gene expression profiles suggesting that O11 and O46 respond to these growth conditions in distinct ways (Table S3, S4, S5).

Whatever the growth phase, O46 overexpressed genes encoding surface components, e.g. *cap* operon or adhesin genes (*fnbB* and *clfA*), in addition to other genes such as *clpP* and phage genes, whereas O11 overexpressed genes encoding secreted virulence factors (*hla*, *hlgA*, *scpA*, *splE*) and genes carried by pathogenicity islands as well as genes involved in iron metabolism (*sir* operon, *sbnc*, *isdH*). Overexpression of these latter genes in O11 may account for the higher sensitivity of O11 (compared to O46) to streptonigrin, an antibiotic which is toxic to cells in the presence of intracellular free iron (minimum inhibitory concentration was at least 4-fold higher for O46 than for O11).

O11 and O46 comparison revealed differences in two σ-factors: the *sigS* gene is indeed truncated in O46 (Table S1) and is found transcribed in O11, only, whatever the growth phases (confirmed by RT-qPCR, Table S3); the *rsbU* gene, part of *sigB* operon, is overexpressed in O11 during stationary phase (Table S3). Genome analysis of the two strains revealed that *spoVG* gene is truncated in O11 (Table S1). Both σ_B_ and σ_S_ appear to dramatically differ between O11 and O46. This may have huge consequences considering their central role in gene regulation and, subsequently, virulence expression.

Inasmuch as the accessory gene regulator (*agr*) system is central to the control of virulence gene expression, we specifically tested the *agr* functionality using RT-qPCR targeting *hld* (RNAIII) and *agrA* (RNAII). Both genes are expressed at similar levels in the two strains suggesting that *agr* does not contribute the differences observed between O11 and O46 gene expression profiles.

### Differences in extracellular proteomes between O11 and O46: overproduction of exoproteins by O11

**Figure 3:**
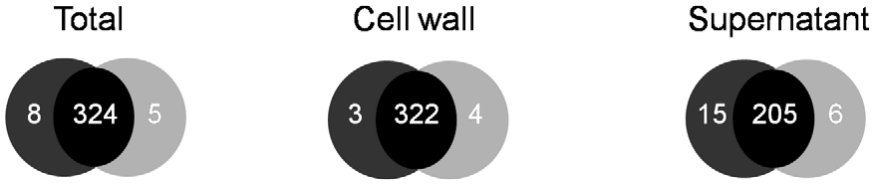
Venn diagram of *S. aureus* O11 and *S. aureus* O46 spots, constructed after analysis of total, cell wall and extracellular fraction 2D-gels with Image Master 2D. Numbers in black-shaded regions represent proteins identified in both O11 and O46 samples. Numbers in dark grey- or light grey-shaded regions indicate proteins specifically identified in *S. aureus* O11 or *S. aureus* O46 respectively. Results are derived from three independent experiments.

Protein samples representing total (whole-cell lysate), cell wall, and extracellular fractions were prepared from O11 and O46 strains grown in conditions identical to those of transcriptomic experiments [32]. At least 3 gels from 3 independent cultures for each strain and each compartment were compared. Image analysis identified 41 spots as being differentially expressed. The majority of differences were observed in extracellular samples, as illustrated in figure 3 (21 spots varied between O11 and O46 in supernatant gels whereas only 20 spots differed in both total and cell wall gels).

It is important to note that each spot may contain more than one unique protein as well as a given protein can be found in several spots. Hereafter, we present data resulting from protein identification (i.e. numbers refer to proteins and not to spots). Protein identification was carried out using Nano-LC analysis and results are listed in Table S6 for extracellular samples, table S7 and S8 (additional files) for total and cell wall samples respectively. Most proteins were common to O11 and O46 as indicated by protein patterns on the 2-D gels from total and cell wall extracts (figure S2 and S3, additional files). Some differences were however observed in both compartments but were mostly due to volume differences and few were due to the absence of the protein in one of the 2 strains. Some proteins were present in both strains but at clearly different positions on the 2-D gels, or they were found in several spots (see table S7), like the alkyl hydroperoxide reductase subunit C (spots T9, T10 on figure S2) or the phosphoglycerate kinase (spot T3 in O46 samples and T4 in O11 samples; figure S2). It should be noted that some spots corresponding to different Mr and/or pI contained the same protein (e.g. spots P7 and P8, or T5, T6 and T12, T13 containing the fructose 1,6-biphosphate aldolase; tables S5 and S7, and figures S1 and S2). In summary, 17 proteins were overproduced by O11 and 8 by O46 in total and cell wall fractions representing proteins from various functional categories including metabolism (14), cellular processes and signalling (4), information storage and processing (5) and unknown functions (2).

**Figure 4:**
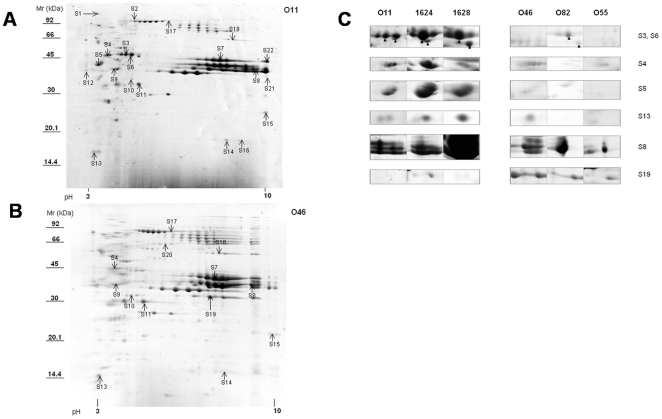
Comparison of exoproteins produced by *S. aureus* strains isolated from clinical or subclinical mastitis. Culture supernatants were harvested after growth in deferoxamine-RPMI during 24 h. 2-DE comparison was carried out by image analysis with Image Master 2D. Spots corresponding to differentially produced protein(s) are indicated by arrows and numbers (S1 to S22). Protein identification was carried out using NanoLC MS/MS (see Table S5 for details). A: a representative 2-DE gel of *S. aureus* O11 secreted proteins, B: a representative 2-DE gel of *S. aureus* O46 secreted proteins, C: production of 6 protein spots in 6 different strain supernatants are depicted with their spot. Strains O11, 1628, and 1624 were isolated from clinical mastitis cases; Strains O46, O82, and O55 were isolated from subclinical mastitis cases. Mr: Molecular weight marker.

In contrast, the extracellular proteomes revealed more pronounced differences (figure 4A and 4B, and additional files table S6, S8, figure S4). A majority of proteins (28 out of the 35 proteins that differed between O11 and O46) were overproduced in O11 extracellular 2-D gels compared to O46. They are directly implicated in virulence (32%; e.g. LukE, LukM, Hla, or Hlg, or Sbi), or predicted to play a role in metabolism or other cellular processes (e.g. IsdA,B,C, and H, involved in iron metabolism). Surprisingly, when considering the predicted location of the proteins (according to the SurfG+ analysis of O11 and O46 genome sequences), many (43%) are predicted to be cytoplasmic (e.g. GAPDH, CspA, or PurH).

Some differences in protein expression can be explained by the presence of indels in one of the 2 strains, which most likely result in a difference in transcriptome and proteome. This is the case for IsdH, whose gene contains a deletion of 1215 bp in O46, and IsdA, whose gene contains an insertion of 45 bp in O46. For some proteins, transcriptomic results (overexpression of the corresponding genes) corroborated proteomic results as shown for Asp23, 2,3-bisphosphoglycerate-dependent phosphoglycerate mutase, IsaA, DapA that are overexpressed by O46 or general stress protein 20 U, ClpL, Hla, mercury(II) reductase that are overexpressed by O11. Some proteins are overexpressed by one strain in log phase and by the other one during stationary phase. For instance, *lukE* and *nuc* are overexpressed in log phase at transcriptomic and proteomic levels (data not shown) by O46 but appeared to be overproduced by O11 during late stationary phase at proteomic level (S8 and S15 figure 4 and figure S4). Finally the overproduction of many proteins by O11 is not always explained by a difference in genome sequence or by higher gene expression. Post-transcriptional regulation, modifications or stability of proteins may contribute to these differences.

### Some proteins differentially produced by O11 and O46 are distributed among a panel of strains isolated from clinical vs subclinical ewe mastitis

In order to identify protein candidates to characterize strains isolated from clinical versus subclinical mastitis, we screened an additional 4 strains isolated from subclinical (n = 2) and clinical (n = 2) cases of ewe mastitis for the presence of the previously identified proteins by proteome analysis of extracellular samples (2-D gels and Coomassie blue staining). Twenty two proteins that were identified as differentially produced by O11 and O46 were checked in other strains. At least 7 spots of these proteins appeared to be also overproduced either by clinical strains or by subclinical strains (figure 4C). SspB, AdhA, LdH, Gap, AhpC, SspA, CspA, Hla, LukM, LukF-PV were overproduced by O11, 1624 and 1628 (severe mastitis isolates) whereas O46_2740 gene product (with similarity to exfoliative toxin family) was overproduced by O46, O82 and O55 (subclinical mastitis isolates).

## Discussion

*S. aureus* mastitis outcomes are highly variable and depend, in part, on strain-dependent features. Here we have achieved the first in-depth characterization of 2 *S. aureus* strains that were shown to reproducibly induce different symptoms in experimental mastitis despite close genotypic relatedness [15]. Complementary approaches were used to gain insight in the molecular basis of *S. aureus* virulence variability in mastitis. Taken together, the results show limited divergence in gene content and clear differences in gene expression. The combined results suggest that differences in iron metabolism, transcriptional regulators and exoprotein production capacity may contribute to the differences observed in mastitis severity induced by these two strains.

### Ability to acquire DNA of exogenous origin and mobile genetic elements

Around 15% of the *S. aureus* genome is composed of MGE, i.e. bacteriophages, transposons, plasmids or pathogenicity islands that can be horizontally transferred from one isolate to another. The restriction-modification systems control in part, the uptake of foreign DNA by bacteria, by identifying and modifying specific DNA sequences so as to prevent the uptake of deleterious DNA for the bacteria (lysogenic bacteriophages or superfluous genes). Four restriction-modification systems have been described in *S. aureus* [30]. The enhanced ability of *S. aureus* strain RN4220 to accept foreign DNA is due to frameshift mutations in *hsdR* gene, a gene belonging to a type 1 restriction-modification system [30] and in a gene encoding a type III-like restriction endonuclease [29]. These two genes were found truncated in O11. This likely explains how O11 was amenable to transformation by plasmid DNA directly extracted from *E. coli*, whereas O46, which contains intact restriction modification genes was not. O11 is thus a naturally transformable strain, which can be useful to further study gene function in the pathogenesis of mastitis. *S. aureus* strains that are deficient in these restriction systems are hypersusceptible to the horizontal transfer of DNA [29]. In addition to being highly virulent, O11 strain may become a reservoir of horizontally acquired antibiotic resistance genes. Occurrence and spread of such strains in lifestock thus deserve special attention.

Surprisingly, O46, and not the transformable O11, contains an additional prophage. Furthermore, phage genes are expressed at higher levels in O46 compared to O11. Phages have been shown to play a crucial role in virulence [33]–[35]. The potential role of the additional prophage found in O46 in mastitis pathogenesis has to be further determined. In contrast, O11 overexpressed genes carried by pathogenicity islands although these latters are found in both O11 and O46 strains. Differences in the expression of MGE-related genes between strains O46 and O11 may contribute to the relative pathogenic potential of the 2 strains.

### Dramatic differences between O11 and O46 gene expression profiles with regard to iron acquisition and metabolism

Iron is an absolute requirement for the growth of most microorganisms and serves as a cofactor in many enzymatic reactions and as a catalyst in electron transport processes [36]. It is however present at a very low concentration in many environments (e.g. in milk, the concentration of available iron is around 10^−12^ µM) [37]. Bacteria have developed various mechanisms to overcome iron restriction [38] and *S. aureus* is able to grow in the presence of extremely low (0.04 µM) iron concentrations [39]. Growth of strains O11 and O46 in deferoxamine-RPMI was carried out to mimic nutritional deficiencies relevant to the mammary gland. Differences regarding genes involved in iron acquisition were revealed through genome, transcriptome and proteome analyses. Indeed, O46 contains truncated genes related to different systems involved in iron metabolism (*isdH*, *hrtB* and *feoA*). Moreover, several genes involved in iron uptake (*sir* operon, some genes of *isd* operon, *fer*, *sstA*, *sbnC*) were overexpressed in O11. Such overexpression was confirmed at the proteomic level for some gene products, like IsdA, B, C, H, that were found overproduced by O11.

The *S. aureus* requirements for iron during infection can be satisfied through several different systems. Heme acquisition by the Isd system is required for full virulence in several models of pathogenesis [40]. It involves 9 proteins, 4 of which were found truncated (*isdH*) and or differentially expressed (*isdA*, *isdB*, *isdC*, and *isdH*) in strain O46. High intracellular concentrations of heme are toxic and *S. aureus* possesses de-toxification systems such as the HrtAB system, a hemin-regulated ABC transporter that protects *S. aureus* against hemin toxicity [41]. In O46, *hrtB* gene is truncated and it appears that the hemin uptake (*isd* system) and detoxification (*hrtAB*) pathways are attenuated in O46. In addition to its role in heme uptake, IsdH was shown to inhibit complement binding at the cell surface and so to contribute to the host immune evasion [42]. Interruption of *isdH* induced a reduced virulence in a mouse sepsis model [42] and a truncated *isdH* in O46 may take part in the reduced severity observed in ewe mastitis as well. Other iron uptake systems differ between O11 and O46. Iron can be acquired in its ferrous form via the widespread bacterial FeoAB system or via siderophore through the SstABCD, SirABC, FhuBCD systems. It is worth noting that *feoA* is truncated in O46 whereas several siderophore genes are overexpressed in O11. Notably *sbnC*, a gene encoding staphylobactin, an *S. aureus* siderophore and *sirABC*, the genes encoding its cell receptor are overexpressed in O11. It has been shown that siderophore production enhances the virulence of *S. aureus* [38]. *S. aureus* strains isolated from bovine mastitis have been shown to be able to overcome iron starvation [43]. However, the underlying mechanisms may vary between human and ruminant isolates. Genes involved in iron uptake or metabolism may reflect host tropism, as suggested by allelic variations reported in these genes in the recently released genome sequences of bovine and ovine isolates [24], [28]. O11 was more sensitive to streptonigrin when grown on deferoxamine-RPMI which suggests, together with higher expression of siderophore genes, that it has a better iron acquisition compared to O46. Strain-dependant expression of genes involved in iron acquisition or metabolism may impact on the severity of infection *in vivo*, in the mammary gland.

### Overexpression of exoproteins by O11

Transcriptomic and proteomic comparison revealed that O11 overexpressed genes encoding exoproteins whereas O46 overexpressed genes encoding surface components. Notably, proteomic analysis of the culture supernatants revealed differences in toxin and protease expression. This was confirmed by the analysis of extracellular fraction in 4 additional strains. This global trend was also observed at the transcriptomic level but the difference in exoprotein levels did not always correlate with transcription implying differences in mRNA half-life or post-translational regulation. It is worth noting that some genes involved in the *sec* pathway (*spsB* and *4.5S RNA*) and genes of the accessory *sec* system (*secY2* and *asp21*) are overexpressed in O11 but a link between expression of these genes and the overproduction of exoproteins has still to be demonstrated.

Of note, differences were observed between the two strains with regard to transcriptional regulators. The *sigS* gene (σ_S_ plays a role in virulence *in vivo* [44]) is truncated in O46 whereas it is intact and transcribed in O11. The *rsbU* gene (part of *sigB* operon) is overexpressed in O11 compared to O46 (Table S3). Consequently in O11, one would expect an up-regulation of σ_B_ regulon including surface components like *cap* operon and surface proteins and a down-regulation of many toxins and secreted proteases [45]. Yet we observed the opposite situation in O11, and further found that the *spoVG* gene, part of *yabJ-spoVG* locus, a σ_B_ effector that modulates σ_B_ control over its dependent genes lacking an apparent σ_B_ promoter, is truncated in O11 (Table S1). This correlates well with the lower nuclease activity observed in O11, as expected according to [46] (Figure S5, supplemental data). Altogether, these differences might account for the differences in expression levels of genes encoding exoproteins (and putatively in mastitis severity) observed for the two strains [15]. Among the oversecreted proteins in O11, some candidates are indeed of special interest and make sense when considering the mastitis context. LukM/F' has been reported to be produced at higher levels during severe mastitis than in moderate mastitis [18], [19]. Production of α hemolysin has also been reported to play a role in mastitis severity [47] and to be involved in gangrenous mastitis [16], [17].

SspB and SspA belong to a proteolytic cascade where a metalloprotease aureolysin (Aur) activates a serine protease zymogen proSspA, which in turn activates the SspB cysteine protease [48]. SspA and SspB play an important role in virulence in a mouse abscess model [49] and they are both involved in the degradation of conjonctive tissue [50]. SspB plays a role in local inflammation of the tissue [51] and in blocking phagocytosis by neutrophils and inhibiting their chemotactic activity [52]. SspA and SspB have also been reported to be produced *in vivo* during gangrenous mastitis [15]. Other exoproteins (e.g. Gap, CspA, AhpC) were found overproduced in the extracellular fraction of O11 and 2 additional strains isolated from severe mastitis although they were predicted to be cytoplasmic. Gap has been reported to be surface located and able to bind to bovine transferrin, which is another high-affinity iron-scavenging mechanism [53]. CspA, a general stress protein is also a strong antigen in human sepsis caused by *S. aureus* [54]. AhpC is not required for virulence *in vivo* but plays a role in nasal colonization and has an important role in host-pathogen interaction [55]. Except Gap, these proteins have not previously been reported to be produced by mastitis *S. aureus* isolates. These three proteins have been reported to be present in *S. aureus* supernatant in other studies [56], [57] but are predicted to be localized in the cytoplasm. A new secretion system involved in protein export via vesicule secretion has recently been described [58]. The secretion mechanism of these proteins is still unknown but this may play a role in the oversecretion of some proteins by O11.

Only one protein was shown to be specifically associated to *S. aureus* strains isolated from moderate mastitis. This analog of exfoliative toxin D has also been shown to be produced *in vivo* [15]. Interestingly, a highly similar exfoliative toxin (76% homology) is also produced by coagulase negative staphylococci, which are the predominant pathogens responsible for subclinical mastitis. Whether this newly identified toxin is active and plays a role in mastitis remains to be determined.

These results clearly show that some exoproteins are specifically produced by isolates associated with severe mastitis. Proteins, like LukM/F' or α hemolysin, have been previously reported to be associated with severe mastitis. To our knowledge, this is the first time a link between proteins other than LukM/F' and Hla and mastitis severity is suggested. These proteins are thus good candidates for further investigation of their exact role in mastitis onset and severity.

### Conclusion

The current study provides the first high resolution comparison of gene content and expression for *S. aureus* mastitis isolates from ovine origin. The results indicate several systems that may contribute to mastitis severity, including MGE, iron metabolism, sigma regulators and exoprotein production. These pathways represent excellent candidates for targeted studies of the molecular basis of *S. aureus* pathogenesis in ruminant mastitis.

## Materials and Methods

### Bacterial strains, growth conditions

*Staphylococcus aureus* O46 was isolated from a case of ovine subclinical mastitis and O11 from a lethal gangrenous mastitis [25]. *S. aureus* O46 and O11 are representative of the major lineage found associated to ewe mastitis in southeast of France [27], [59]. Four other *S. aureus* strains isolated from gangrenous and clinical ewe mastitis (1628 and 1624, respectively) and subclinical ewe mastitis (O55 and O82) were used in this study and were previously described [27]. Growth conditions and preparation of protein extracts were as described in Le Maréchal et al. 2009 [32]. Briefly, all cultures were performed as follows: Overnight cultures in BHI were diluted 1∶1000 in fresh RPMI 1640 medium (Sigma, Saint Quentin fallavier, France). Deferoxamine (0.15 mM; Sigma), an iron chelator, was added to RPMI (hereafter referred to as deferoxamine-RPMI). *S. aureus* strains were grown anaerobically in falcon tubes (50 ml) or in flasks (250 ml) filled up with medium and incubated at 37°C without agitation in anaerobic conditions. The same anaerobic conditions were used to compare O11 and O46 transcriptome and proteome. Minimum inhibitory concentration for streptonigrin (Sigma) was determined as follows. Overnight cultures of O11 and O46, on BHI, were 1/100 diluted and used to inoculate fresh deferoxamine-RPMI (2.5 mL, in 15-mL tubes) containing increasing concentrations of streptonigrin (0, 1.25, 2.5, 5, 10, and 20 ng/mL). Cultures were incubated at 37°C under agitation.

### RNA, DNA and protein extraction

Protein samples for extracellular, cell wall or total fraction, RNA extraction and purification and genomic DNA extraction were exactly done as previously described [32], [59], [60].

### Genome sequencing, assembly, annotation and comparison of O11 and O46

Whole genome sequencing and assembling strategy are described in [23]. Comparison and graphical mapping were performed using the MUMmer software package [61], the Circos visualization software [62] as well as an application developed in house. Coding sequences (CDSs) detection was performed with the Glimmer software application [63]. Annotations were imported from already annotated *S. aureus* strains and mapped to the corresponding CDSs by using an application developed in house as well as the Exonerate sequence alignment program [64]. These genome sequences have been deposited at DDBJ/EMBL/GenBank under the accession AEUQ00000000 (O11) and AEUR00000000 (O46) [23].

### Microarray design and manufacturing

The microarray was manufactured by *in situ* synthesis of 60-base oligonucleotide probes (Agilent, Palo Alto, CA), selected as previously described [65]. The array covers 98% of all open reading frames (ORFs) annotated in strains N315, Mu50, COL, MRSA252, MSSA476, MW2, USA300_FPR3757, NCTC8325, RF122 including their respective plasmids.

### Preparation of labeled nucleic acids for expression microarrays

Total RNA was purified from bacteria grown in deferoxamine-RPMI during log phase (OD_600_ = 0.5) and stationary phase (OD_600_ = 1). For each strain total RNA of three independent cultures was extracted as previously described [60]. After additional DNase treatment, noncontamination of the RNA sample by genomic DNA (gDNA) was confirmed by quantitative PCR on *gyrB*. Batches of 8 µg of total *S. aureus* RNA were labeled with Cy3-dCTP using SuperScript II (Invitrogen, Basel, Switzerland) following the manufacturer's instructions. Labeled products were then purified onto QiaQuick columns (Qiagen). The following steps were performed as described in [66].

### Microarray analysis

Fluorescence intensities were extracted using Feature Extraction software (version 8; Agilent). Local background-subtracted signals were corrected for unequal dye incorporation or unequal load of the labelled product. Per chip normalizations were performed using the 50th percentile of all measurements for different hybridisations to make comparisons between different experiments valid. Data consisting of three independent biological experiments were analyzed using GeneSpring, version 8.0 (Silicon Genetics, Redwood City, CA) after per gene and per chip normalization. Statistical significance of differential gene expression was calculated by analysis of variance using GeneSpring, including the Benjamini and Hochberg false discovery rate correction of 5% (P value cutoff, 0.05) and higher than 2-fold induction or reduction of expression was accepted as differential expression.

### Microarray data accession number

The microarray design and the complete dataset were deposited in the public repository database Gene Expresion Omnibus under the accession numbers GPL11137 and GSE25084, respectively.

### qRT-PCR

To confirm microarray data, expression profiles of *clfA*, *sigS*, *sirA*, *urea*, *hld*, *ahpF*, *phoP*, *agrA*, *capA* were determined by quantitative reverse transcription-PCR (qRT-PCR) analyses. Primer sequences are given in Table S9. All primer efficiencies were tested for each strain and ranged between 85% and 110%. cDNA was synthesized using the high-capacity cDNA archive kit as recommended by the manufacturer (Applied Biosystems, Warrington, United Kingdom). Quantitative real-time PCR was performed using an Opticon 2 real-time PCR detector (Bio-Rad, Hercules, CA). The reaction mixture contained power Sybr green PCR master mix (1X; Applied Biosystems, Warrington, United Kingdom), each primer (0.5 µM), and 1 µg cDNA template. Thermal cycling consisted of 10 min at 95°C, followed by 40 cycles of 15 s at 95°C and 60 s at 60°C. qRT-PCR analyses for all experimental time points were performed in triplicate (using three independent biological replicates). Calibration curves were generated to calculate the copy number for each gene in each sample. *gyrA*, *ftsZ*, *hu* and *sodA* [67] were tested to determine the best internal standards for normalization using the geNorm VBA applet for Microsoft Excel. *gyrA*, *ftsZ* and *sodA* were used as internal standards for exponential phase and *ftsZ* and *sodA* for stationary phase. The Ct values of genes of interest were transformed to quantities (number of copies) by using standard curves. Gene expression levels were calculated by dividing gene of interest quantities by the previously calculated normalization factor (according to the geNorm user manual). Statistical analyses were performed as in [68].

### 2-Dimensional Gel Electrophoresis

Protein samples (200 µg) were precipitated with 2D clean up kit (GE Healthcare, Orsay, France) according to the manufacturer's instructions. Pellets were solubilised in sample solution containing 7 M urea, 2 M thio-urea, 25 mM dithiothreitol (DTT), 4% (w/v) 3-[(3-Cholamidopropyl)dimethylammonio]-1-propane-sulfonate (CHAPS) and 2% (w/v) ampholyte containing buffer (IPG-Buffer 4–7 or 3–10 NL, GE Healthcare). Isoelectric focusing was carried out using pH 4 to 7 (Cell wall and total proteins) or 3 to 10 NL (extracellular fraction) 13 cm Immobiline Dry Strips on a Multiphor II electrophoresis system (Amersham Biosciences) as described previously [15]. The second dimension separation was performed on an Ettan dalt electrophoresis system (GE Healthcare) using 14% acrylamide separating gels without a stacking gel at a voltage of 50 V for 1 h and 180 V for 7 h. Low molecular weight markers (GE Healthcare) were used as the standards. Gels were stained with R250 Coomassie blue (Serva, Heildelberg, Germany). Three extractions from three different cultures were carried out to perform 2-D gels. Stained 2-D gels were scanned with Image Scanner II (Amersham biosciences) and image analysis was performed with ImageMaster 2D platinum software as previously described [69], [70].

### Nano-LC analysis

Proteins were identified by tandem mass spectrometry (MS/MS) after an in-gel trypsin digestion adapted from Shevchenko [71] and described in details in [15]. Briefly, gel pieces were excised from the gel. In-gel trypsin digestion was performed overnight at 37°C. After concentration, the supernatants containing peptides were analyzed using an on-line liquid chromatography tandem mass spectrometry (MS/MS) setup. A full continuous MS scan was carried out followed by three data-dependent MS/MS scans. Spectra were collected in the selected mass range 400 to 2,000 *m/z* for MS and 60 to 2,000 *m/z* for MS/MS spectra. The three most intense ions from the MS scan were selected individually for collision-induced dissociation (1+ to 4+ charged ions were considered for the MS/MS analysis). The mass spectrometer was operated in data-dependent mode automatically switching between MS and MS/MS acquisition. The proteins present in the samples were identified from MS and MS/MS data by using MASCOT v.2.2 software for search into two concatenated databases: (i) a homemade database containing all the predicted proteins of the O11 and O46 strains used in this study as deduced from their genome [23] and (ii) a portion of the UniProtKB database corresponding to the *Staphylococcus aureus* taxonomic group (http://www.uniprot.org/).

## Supporting Information

Table S1 Truncated genes in *S. aureus* O11 or *S. aureus* O46 sequences.
(DOCX)

Table S2 Amenability of O11 and O46 strains to transformation.
(DOC)

Table S3 Genes discussed in this work that were differentially expressed during log and stationary phases between O11 and O46.
(DOCX)

Table S4 Expression profiles of genes exhibiting significant variations between O11 and O46 during log phase in deferoxamine-RPMI medium.
(DOC)

Table S5 Expression profiles of genes exhibiting significant variations between O11 and O46 during stationary phase in deferoxamine-RPMI medium.
(DOC)

Table S6 *S. aureus* O11 and *S. aureus* O46 extracellular proteins identified in this study.
(DOCX)

Table S7 Proteins identified by nanoLC MS/MS as being differentially produced by O11 or O46 after analysis of 2D gels of total cell lysate samples (figure S2).
(DOC)

Table S8 Proteins identified by nanoLC MS/MS as being differentially produced by O11 and O46 after analysis of 2D gels of cell wall extract (figure S2).
(DOC)

Table S9 Oligonucleotides used in this study for quantitative real-time PCR.
(DOC)

Figure S1 Biofilm production in *S. aureus* O11 and O46 as determined by Cristal violet staining assay. Biofilm staining assays were performed as described previously [29]. Briefly, after bacterial growth in iron-depleted RPMI, microtiter plates (MultiwellTM 6 well, Becton Dickinson) were washed twice with phosphate-buffered saline (PBS), fixed for 20 min at 80°C and stained for 10 min with 1% (w/v) crystal violet solution freshly diluted twofold in 1% (v/v) ethanol/distilled water. Plates were then washed with water and photographed. The crystal violet was dissolved in dimethyl sulfoxide (DMSO) for 1 h before OD_600 nm_ measurements. Biofilm formation was estimated for each strain, on 6 replicates, and the data were analysed by the student's paired *t* test. A *P* value of 0.05 or less (here, *P* = 0.044).was considered statistically significant.
(TIF)

Figure S2 Proteomic comparison of *S. aureus* O11 and *S. aureus* O46 cell lysates. A: representative 2-DE gel of *S. aureus* O11 (upper gel) and *S. aureus* O46 (lower gel) total lysates. Proteins were prepared after growth in iron-depleted RMPI. 200 µg of protein preparation was separated on 13 cm gels (pI 4–7, 14% SDS-PAGE) and Coomassie Blue-stained. Image analysis with Image Master 2D revealed differences in the protein spots indicated with arrows and numbers. Identification was carried out by NanoLC MS/MS (see Table S7). B: The expression of numbered spots are depicted in three different gels prepared from three biological replicates (R1, R2, R3) from O11 or O46 total lysates.
(TIF)

Figure S3 Proteomic comparison of *S. aureus* O11 and *S. aureus* O46 cell wall proteins. A: representative 2D gel of O11 (upper gel) and O46 (lower gel) cell wall extracts. Proteins were prepared after growth in iron-depleted RMPI. 200 µg of protein preparation was separated on 13 cm gels (pI 4–7, 14% SDS-PAGE) and Coomassie Blue-stained. Image analysis with Image Master 2D revealed differences in the protein spots indicated with arrows and numbers. Identification was carried out by NanoLC MS/MS (see Table S8). B: The expression of numbered spots are depicted in three different gels prepared from three biological replicates (R1, R2, R3) from O11 or O46 cell wall samples.
(TIF)

Figure S4 Proteomic differences between *S. aureus* O11 and *S. aureus* O46 extracellular proteins highlighted by image analysis with Image master 2D. Identification was carried out by NanoLC MS/MS (see Table S6). The expression of numbered spots are depicted in three different gels prepared from three biological replicates (R1, R2, R3) from O11 (left panel) or O46 (right panel) extracellular protein samples.
(TIF)

Figure S5 Nuclease activity assay on supernatant of O11 and O46. A nuclease plate assay was carried out on supernatant of O11 and O46 strains after overnight culture on deferoxamine-RPMI. 10 µL of 0.2 µm filtered supernatant were spotted on Toluidine Blue-DNA agar as described previously [72]. Plates were incubated o.n. at 37°C and nuclease activity was revealed by the development of a pink halo, which diameter is proportional to the amount of Nuclease secreted. Presence of truncated *spoVG* in O11 correlates with a lower nuclease production as previously reported [46].
(TIF)
