# Supplementary material for: Molecular Basis of Virulence in Staphylococcus aureus Mastitis
Source: PLoS One. 2011 Nov 11;6(11):e27354. doi: 10.1371/journal.pone.0027354 (PMC3214034; doi:10.1371/journal.pone.0027354)
Supplement: Table S1 — Truncated genes in S. aureus O11 or S. aureus O46 sequences. (DOCX) [file pone.0027354.s001.docx]

**Table S1: Truncated genes in *S. aureus* O11 or *S. aureus* O46 sequences.**

| **Gene^1^** | **Description^2^** | O11 CDS ^3^ | **L^4^** | **O46 CDS^3^** | **L^4^** | **Loca^5^** | **Trans^6^** | **Prot^7^** |
| --- | --- | --- | --- | --- | --- | --- | --- | --- |
| **INFORMATION STORAGE AND PROCESSING** |  |  |  |  |  |  |  |  |
| **Transcription** |  |  |  |  |  |  |  |  |
| *sigS* | DNA-binding protein | 011_1876 | 549 | 046_2447 | 258 | C | X |  |
| *rpoE* | DNA-directed RNA polymerase subunit delta | 011_2038 | 516 | 046_0924 | 531 | C |  |  |
| --- | TetR family regulatory protein | 011_0166 | 588 | 046_0168 | 522 | C | X |  |
| *tenA* | transcriptional activator | 011_2361 | 663 | 046_1834 | 690 | C |  |  |
| --- | LysR family regulatory protein | 011_0305 | 249 | 046_0375 | 870 | C |  |  |
| --- | transcriptional regulator | 011_1427 | 597 | 046_1416 | 2238 | C |  |  |
| --- | transcriptional regulator | 011_0428 | 354 | 046_2354 | 336 | C |  |  |
| --- | acetyltransferase | 011_2639 | 288 | 046_0440 | 552 | C |  |  |
| **Replication, recombination and repair** |  |  |  |  |  |  |  |  |
| --- | DNA damage repair protein | 011_1021 | 1233 | 046_0866 | 1263 | C | X |  |
| *radC* | DNA repair protein RadC | 011_1319 | 441 | 046_0617 | 672 | C |  |  |
| *hsdR* | type I restriction enzyme restriction chain | 011_0749/O11_0748 | 2775 | 046_0485 | 2790 | C |  | X |
| --- | putative helicase, Similar to Type III restriction protein res subunit | 011_0246/011_0247/011_0248 | 2814 | 046_2610 | 2862 | C |  | X |
| --- | TOPRIM domain-containing protein | 011_2700 | 393 | 046_2509 | 390 | C |  |  |
| *polA* | phage related DNA polymerase family A | 011_1630 | 1953 | 046_2661 | 678 | C | X |  |
| --- | putative insertion element protein | 011_1224 | 687 | 046_0005 | 1230 | C |  |  |
| **CELLULAR PROCESSES AND SIGNALING** |  |  |  |  |  |  |  |  |
| **Cell cycle control, cell division, chromosome partitioning** |  |  |  |  |  |  |  |  |
| --- | SpoIIIE family cell division protein | 011_1915 | 3834 | 046_1342 | 3825 | C |  |  |
| **Cell wall/membrane/envelope biogenesis** |  |  |  |  |  |  |  |  |
| *spoVG* | regulatory protein SpoVG | 011_1367 | 291 | 046_2417 | 303 | C |  |  |
| --- | LysM domain-containing protein | 011_2589 | 840 | 046_2219 | 393 | PSE | X |  |
| **Signal transduction mechanisms** |  |  |  |  |  |  |  |  |
| --- | sensor kinase protein | 011_0978 | 849 | 046_0823 | 843 | M |  |  |
| **Defense mechanisms** |  |  |  |  |  |  |  |  |
| --- | FmhC protein | 011_0899 | 768 | 046_0744 | 606 | C |  |  |
| --- | ABC transporter ATP-binding protein | 011_2632 | 642 | 046_0432 | 312 | C |  |  |
| --- | secretory antigen precursor | 011_0580 | 804 | 046_1136 | 813 | S | X |  |
| --- | membrane anchored protein | 011_1762 | 1176 | 046_1982 | 1215 | PSE |  |  |
| --- | putative enterotoxin type A | 011_2666 | 753 | 046_2274 | 705 | C |  |  |
| *sasA* | Serine-rich adhesin for platelets | 011_1100 | 5337 | 046_1536 | 6582 | PSE |  |  |
| *scpA* | staphopain cysteine proteinase | 011_1718 | 1140 | 046_2362 | 1167 | S | X |  |
| --- | surface protein | 011_1325 | 684 | 046_1386 | 675 | PSE | X |  |
| *vwb* | von Willebrand factor-binding protein | 011_2067 | 1518 | 046_2531 | 1524 | PSE |  |  |
| *set5* | superantigen-like protein 7 | 011_1523 | 120 | 046_0938 | 114 | C |  |  |
| *set2* | superantigen-like protein | 011_1520/011_1519 | 670 | 046_0935 | 684 | S |  |  |
| **METABOLISM** |  |  |  |  |  |  |  |  |
| **Energy production and conversion** |  |  |  |  |  |  |  |  |
| --- | Appr-1-p processing domain-containing protein | 011_1794 | 801 | 046_1587 | 792 | C | X |  |
| --- | glycerate dehydrogenase | 011_0355/ 011_0356 | 285 | 046_0459 | 960 | C |  |  |
| **Amino acid transport and metabolism** |  |  |  |  |  |  |  |  |
| *ald* | alanine dehydrogenase | 011_2104 | 1119 | 046_1705 | 141 | C |  |  |
| --- | glutamate synthase-ferredoxin large subunit | 011_2431 | 1275 | 046_2109 | 1578 | PSE |  |  |
| --- | Glutathione S-transferase transmembrane protein | 011_1940 | 357 | 046_0252 | 468 | C |  |  |
| **Nucleotide transport and metabolism** |  |  |  |  |  |  |  |  |
| --- | nucleoside transporter permease | 011_1653 | 366 | 046_0680 | 1221 | M |  |  |
| **Carbohydrate transport and metabolism** |  |  |  |  |  |  |  |  |
| --- | transketolase | 011_0999 | 1974 | 046_0844 | 1989 | C |  | X |
| --- | multiple sugar-binding transport ATP-binding protein | 011_0056 | 516 | 046_0496 | 510 | C |  |  |
| --- | peptidase | 011_1023 | 1032 | 046_0868 | 288 | C |  |  |
| --- | antibiotic resistance-related transmembrane efflux protein (pid:82749819) | 011_1430 | 135 | 046_1414 | 1389 | M |  |  |
| *icaC* | icaC | 011_1116 | 783 | 046_1520 | 1053 | M |  | X |
| *lacC* | tagatose-6-phosphate kinase | 011_1840 | 933 | 046_1691 | 924 | C |  |  |
| **Lipid transport and metabolism** |  |  |  |  |  |  |  |  |
| *accC* | acetyl-CoA biotin carboxylase | 011_2478 | 666 | 046_2279 | 1362 | C |  |  |
| *acsA* | acetyl-CoA synthetase | 011_1245 | 522 | 046_1351 | 1707 | C |  |  |
| **Inorganic ion transport and metabolism** |  |  |  |  |  |  |  |  |
| --- | high-affinity nickel-transport protein | 011_1147 | 126 | 046_1490 | 1053 | M |  |  |
| --- | lipoprotein | 011_0770 | 441 | 046_0735 | 975 | PSE |  |  |
| --- | DMT superfamily drug/metabolite transporter | 011_0204 | 753 | 046_0129 | 963 | M | X |  |
| **Iron metabolism** |  |  |  |  |  |  |  |  |
| *isdH* | isdH | 011_1248 | 2682 | 046_1353 | 2697 | PSE | X | X |
| *feoA* | ferrous iron transport protein A | 011_0187 | 228 | 046_0146 | 168 | C |  |  |
| *hrtB* | Putative hemin transport system permease protein hrtB | 011_0645 | 1056 | 046_1071 | 975 | PSE |  |  |
| **Coenzyme transport and metabolism** |  |  |  |  |  |  |  |  |
| --- | 4'-phosphopantetheinyl transferase superfamily protein | 011_0764 | 645 | 046_0470 | 564 | C |  |  |
| **POORLY CHARACTERIZED** |  |  |  |  |  |  |  |  |
| **General function prediction only/ Function unknown** |  |  |  |  |  |  |  |  |
| --- | acetyltransferase | 011_2582 | 360 | 046_2226 | 282 | C |  |  |
| --- | Antibiotic biosynthesis monooxygenase | 011_0127 | 420 | 046_0206 | 480 | C | X |  |
| --- | competence-related protein | 011_0285 | 936 | 046_0398 | 987 | C |  |  |
| --- | hypothetical protein | 011_0036 | 345 | 046_0517 | 147 | C |  |  |
| --- | hypothetical protein | 011_0025 | 357 | 046_0527 | 255 | C |  |  |
| --- | hypothetical protein | 011_0228 | 1212 | 046_0105 | 1824 | PSE |  |  |
| --- | hypothetical protein | 011_0347 | 339 | 046_0333 | 234 | C |  |  |
| --- | hypothetical protein | 011_0976 | 894 | 046_0822 | 1608 | PSE |  |  |
| --- | hypothetical protein | 011_1008 | 297 | 046_0853 | 252 | C |  |  |
| --- | hypothetical protein | 011_1041 | 351 | 046_0888 | 216 | M |  |  |
| --- | hypothetical protein | 011_1140 | 768 | 046_1497 | 816 | M |  |  |
| --- | hypothetical protein | 011_1333 | 735 | 046_1394 | 762 | PSE |  |  |
| --- | hypothetical protein | 011_1397 | 741 | 046_1446 | 651 | C |  |  |
| --- | hypothetical protein | 011_1646 | 1362 | 046_0687 | 111 | C |  |  |
| --- | hypothetical protein | 011_1690 | 1524 | 046_0644 | 1386 | C | X |  |
| --- | hypothetical protein | 011_2409 | 441 | 046_2056 | 435 | PSE |  |  |
| --- | hypothetical protein | 011_0059 | 87 | 046_0942 | 135 | C |  |  |
| --- | hypothetical protein | 011_0250 | 180 | 046_2607 | 1356 | C |  |  |
| --- | hypothetical protein | 011_0257 | 408 | 046_2601 | 423 | PSE |  |  |
| --- | hypothetical protein | 011_0491 | 369 | 046_2740 | 843 | S |  | X |
| --- | hypothetical protein | 011_1064 | 1926 | 046_0563 | 756 | C |  |  |
| --- | hypothetical protein | 011_1400 | 408 | 046_1444 | 840 | C |  |  |
| --- | hypothetical protein | 011_1755 | 444 | 046_1975 | 345 | M |  |  |
| --- | hypothetical protein | 011_2318 | 1260 | 046_1863 | 2178 | C |  |  |
| --- | hypothetical protein | 011_2351 | 9378 | 046_2560 | 8091 | S |  |  |
| --- | hypothetical protein | 011_2536 | 4113 | 046_2534 | 789 | C |  |  |
| --- | hypothetical protein | 011_2755 | 87 | 046_2338 | 147 | C |  |  |
| --- | hypothetical protein | 011_0862 | 819 | 046_2567 | 939 | C |  |  |
| --- | hypothetical protein | 011_1220 | 87 | 046_0003 | 162 | C |  |  |
| --- | hypothetical protein | 011_1642 | 111 | 046_0691 | 171 | C |  |  |
| --- | hypothetical protein | 011_1817 | 87 | 046_1610 | 90 | C |  |  |
| --- | hypothetical protein | 011_0095 | 573 | 046_0979 | 267 | PSE |  |  |
| --- | hypothetical protein | 011_0675 | 696 | 046_2494 | 546 | S |  |  |
| --- | hypothetical protein | 011_0716 | 660 | 046_1949 | 675 | M | X |  |
| --- | hypothetical protein | 011_0863 | 192 | 046_2568 | 279 | M |  |  |
| --- | hypothetical protein | 011_1149 | 807 | 046_1489 | 318 | C |  |  |
| --- | hypothetical protein | 011_1217 | 1476 | 046_0001 | 1893 | C |  |  |
| --- | hypothetical protein | 011_1407/011_1406 | 2538 | 046_1437 | 813 | C |  |  |
| --- | hypothetical protein | 011_1976 | 813 | 046_0288 | 777 | C |  |  |
| --- | hypothetical protein | 011_2016 | 150 | 046_2464 | 153 | C | X |  |
| --- | metallo-beta-lactamase superfamily protein | 011_1909 | 885 | 046_1335 | 489 | C |  |  |
| --- | N-acyl-L-amino acid amidohydrolase | 011_0404 | 933 | 046_1767 | 1176 | C |  |  |
| --- | NADH-dependent dehydrogenase | 011_0048 | 1041 | 046_0504 | 1029 | C |  |  |
| --- | oxidoreductase | 011_2707 | 801 | 046_2031 | 768 | C |  |  |
| --- | phiSLT ORF 563-like protein, terminase, large subunit | 011_1827/011_1826 | 1221 | 046_1619 | 1692 | C |  |  |
| --- | phiSLT ORF144-like protein, putative lipoprotein | 011_1641 | 384 | 046_0692 | 435 | PSE | X |  |
| --- | phiSLT ORF412-like protein, portal protein | 011_1825 | 1239 | 046_1618 | 1047 | C |  |  |
| --- | phiSLT ORF66-like protein | 011_2617 | 201 | 046_2680 | 132 | C | X |  |
| *pknB* | protein kinase | 011_0869 | 2001 | 046_2574 | 1995 | PSE |  |  |
| --- | Putative membrane protein | 011_2587 | 189 | 046_2221 | 237 | M | X |  |
| --- | *Staphylococcus aureus* phage phi 13 | 011_1193 | 1395 | 046_2017 | 1365 | C |  |  |
| --- | tandem lipoprotein | 011_1425 | 123 | 046_1418 | 771 | PSE |  |  |

^1^: Genes are classified in GO functional classes.

^2^: Names are given according to annotation of available *S. aureus* sequence genomes.

^3^: Coding sequence numbers corresponding to genes in *S. aureus* O11 and *S. aureus* O46

^4^: Gene sequence length

^5^: Predicted protein localization (SurfG+). C, cytoplasmic; S, secreted; PSE, predicted surface exposed

^6^: Differences between *S. aureus* O11 and *S. aureus* O46 confirmed at the transcriptome level

^7^: Differences between *S. aureus* O11 and *S. aureus* O46 confirmed at the proteome level
