# Supplementary material for: Molecular Basis of Virulence in Staphylococcus aureus Mastitis
Source: PLoS One. 2011 Nov 11;6(11):e27354. doi: 10.1371/journal.pone.0027354 (PMC3214034; doi:10.1371/journal.pone.0027354)
Supplement: Table S2 — Amenability of O11 and O46 strains to transformation. (DOC) [file pone.0027354.s002.doc]

**Table S2**: Amenability of O11 and O46 strains to transformation. Plasmidic DNA of pMAD vector (69) was used for transformation test in strains O11, O46 and MW2. S. aureus strains were transformed by electroporation using standard protocols (70) and transformants were selected on BHI plates, with erythromycin (2 µg ml^−1^).

|  | O11 | O46 | MW2 |
| --- | --- | --- | --- |
| pMAD  (extracted from *E. coli* DH5α) | +++ | - | +++ |
| pMAD  (extracted from *S. aureus* O11) | n.d. | + | +++ |
| pMAD  (extracted from *S. aureus* RN4220) | +++ | - | +++ |

+++: transformation efficiency > 10^3^ transformants / µg of plasmidic DNA

+: transformation efficiency <10 transformants / µg of plasmidic DNA

-: no transformants.
