# Supplementary material for: Molecular Basis of Virulence in Staphylococcus aureus Mastitis
Source: PLoS One. 2011 Nov 11;6(11):e27354. doi: 10.1371/journal.pone.0027354 (PMC3214034; doi:10.1371/journal.pone.0027354)
Supplement: Table S3 — Genes discussed in this work that were differentially expressed during log and stationary phases between O11 and O46. (DOCX) [file pone.0027354.s003.docx]

**Table S3: Genes discussed in this work that were differentially expressed during log and stationary phases between O11 and O46.**

| Gene^1^ | **Description^2^** | **O11 CDS^3^** | **O46CDS^3^** | **Expo^4^** | **Stat^4^** |
| --- | --- | --- | --- | --- | --- |
| **Amino Acid metabolism and transport** |  |  |  | - | - |
| *dapA £* | Dihydrodipicolinate synthase | 011_2728 | 046_1043 | 0,45575 | 0,497 |
| **Post-translational modification, protein turnover, chaperone functions** |  |  |  |  |  |
| *clpP* | ATP-dependent Clp protease, proteolytic subunit ClpP | 011_2345 | 046_2232 | 0,461 | - |
| **Transcription** |  |  |  |  |  |
| *glnR* | Glutamine synthetase repressor | 011_0962 | 046_0808 | 0,405 | - |
| *sigS ** | RNA polymerase sigma factor SigS | 011_1876 |  | 2,685 | - |
| *czrA* | Repressor protein | 011_1058 | 046_0570 | - | 0,4375 |
| *rsbU* | SigmaB regulation protein RsbU | 011_2389 | 046_1806 | - | 2,0095 |
| --- | HTH-type transcriptional regulator *sarV* | 011_0548 | 046_1168 | - | 2,659 |
| **iron metabolism genes** |  |  |  |  |  |
| *isdI* | Heme-degrading monooxygenase IsdI | 011_0778 | 046_0727 | - | 0,0976 |
| *isdB £* | Iron-regulated surface determinant protein B | 011_1476 | 046_1296 | - | 0,474 |
| *fer* | Ferredoxin | 011_2322 | 046_1751 | - | 2,032 |
| *isdE* | High-affinity heme uptake system protein isdE | 011_1481 | 046_1291 | - | 2,054 |
| *sstA* | FecCD transport family protein 1 | 011_2504 | 046_1234 | - | 2,313 |
| *sbnC* | Siderophore staphylobactin biosynthesis protein | 011_1342 | 046_1402 | 2,021 | 2,68366667 |
| *sirB* | Iron-regulated ABC transporter siderophore permease protein SirB | 011_1346 | 046_1406 | 3,34 | 3,2075 |
| *sirA ** | Iron-regulated ABC transporter siderophore-binding protein SirA | 011_1345 | 046_1405 | 3,922 | 3,609 |
| *sirC* | Iron-regulated ABC transporter siderophore permease protein SirC | 011_1347 | 046_1407 | 3,146875 | 3,8335 |
| *isdH £* | Iron-regulated surface determinant protein H | 011_1248 | 046_1353 | 7,2851875 | 4,443875 |
| **Intracellular trafficing and secretion** |  |  |  |  |  |
| *secY2* | Preprotein translocase subunit SecY | 011_1099 | 046_1537 | - | 2,291 |
| *spsB* | Type-1 signal peptidase 1B | 011_0317 | 046_0364 | - | 2,847 |
| *asp21* | Accessory secretory protein Asp1 | 011_1098 | 046_1538 | - | 3,054 |
| **defense/virulence factor** |  |  |  |  |  |
| --- | Truncated map-w protein | 011_1748 | 046_2392 | - | 0,476 |
| --- | Protein map | 011_1749 | 046_2393 | - | 0,488 |
| --- | ABC transporter ATP-binding protein 2 | 011_1966 | 046_0278 | - | 2,072 |
| --- | MAP domain-containing protein | 011_0303 | 046_0380 | 3,132 | 2,384 |
| --- | Antibiotic transport system permease | 011_0212 | 046_0121 | - | 2,415 |
| **capsule genes** |  |  |  |  |  |
| *cap8H* | Capsular polysaccharide synthesis enzyme capH | 011_0788 | 046_0717 | 0,304 | 0,43566667 |
| *capD* | Capsular polysaccharide synthesis enzyme CapD | 011_0792 | 046_0713 | 0,373 | 0,42983333 |
| *cap8K* | Capsular polysaccharide synthesis enzyme capK | 011_0785 | 046_0721 | 0,37425 | - |
| *capM* | Capsular polysaccharide synthesis enzyme CapM | 011_0782 | 046_0723 | 0,395 | - |
| *capL* | Capsular polysaccharide synthesis enzyme CapL | 011_0783 | 046_0722 | 0,39575 | - |
| *capN* | Capsular polysaccharide synthesis enzyme CapN | 011_0781 | 046_0724 | 0,423 | - |
| *capP* | Capsular polysaccharide synthesis enzyme CapP | 011_0779 | 046_0726 | 0,423 | 0,463 |
| *cap8I* | Capsular polysaccharide synthesis enzyme capI | 011_0787 | 046_0718 | 0,428 | 0,476 |
| *capF* | Capsular polysaccharide synthesis enzyme CapF | 011_0790 | 046_0715 | 0,447 | 0,4335 |
| *capE* | Capsular polysaccharide synthesis enzyme CapE | 011_0791 | 046_0714 | 0,466 | 0,40644444 |
| *capG* | Capsular polysaccharide synthesis enzyme CapG | 011_0789 | 046_0716 | 0,4765 | 0,424 |
| *capO* | Capsular polysaccharide synthesis enzyme CapO | 011_0780 | 046_0725 | 0,4825 | - |
| *cap8J* | Capsular polysaccharide synthesis enzyme CapJ | 011_0786 | 046_0719 | - | 0,4505 |
| *capP* | Capsular polysaccharide synthesis enzyme CapP | 011_0779 | 046_0726 | - | 0,463 |
| *capA ** | Capsular polysaccharide synthesis enzyme CapA | 011_0795 | 046_0710 | - | 0,4755 |
| *capC* | Capsular polysaccharide synthesis enzyme CapC | 011_0793 | 046_0712 | - | 0,42466667 |
| **adhesion genes** |  |  |  |  |  |
| *fnbB* | FnbB protein |  | 046_2117 | 0,291 | 0,281 |
| *fib* | Fibrinogen-binding protein | 011_1509 | 046_1264 | - | 0,308 |
| --- | Fibrinogen-binding protein-related protein | 011_1510 | 046_1263 | - | 0,434 |
| *clfA ** | Clumping factor A | 011_2325 | 046_2251 | 0,207925 | 0,45 |
| *sdrC* | Ser-Asp rich fibrinogen-binding, bone sialoprotein-binding protein | 011_0417 | 046_1754 | - | 2,003 |
| *sdrD* | Serine-aspartate repeat-containing protein D | 011_2683 |  | 5,1055 | 4,4335 |
| *sdrE* | Serine-aspartate repeat-containing protein E | 011_2763 | 046_2767 | 2,181 | - |
| **antigen genes** |  |  |  |  |  |
| *isaA £* | Immunodominant antigen A | 011_0168 | 046_0166 | - | 0,411 |
| *ssaA2* | Staphylococcal secretory antigen ssaA2 | 011_0580 | 046_1136 | - | 0,463 |
| --- | Secretory antigen SsaA, putative | 011_0584 | 046_1132 | - | 0,3925 |
| *isaB* | Immunodominant antigen B | 011_1084 | 046_1552 | - | 2,638 |
| **enzyme genes** |  |  |  |  |  |
| *splD* | Serine protease SplD | 011_0672 | 046_2496 | 0,4165 | - |
| --- | Staphostatin A superfamily | 011_1719 | 046_2363 | 2,339 | - |
| *scpA* | Staphopain A | 011_1718 | 046_2362 | 3,291 | 2,771 |
| *aur* | Zinc metalloproteinase aureolysin | 011_1083 | 046_1553 | - | 2,092 |
| --- | Staphostatin A superfamily | 011_1719 | 046_2363 | - | 2,372 |
| --- | Probable transglycosylase sceD | 011_2359 | 046_1836 | - | 2,403 |
| --- | Staphylocoagulase precursor | 011_0035 | 046_0518 | - | 3,31 |
| *splF* | Serine protease splF | 011_0672 | 046_2496 | - | 8,899 |
| *splE* | Serine protease splE | 011_0673 |  | 155,4875 | 173,7125 |
| **hemolysin genes** |  |  |  | - | - |
| *hlgA* | Gamma-hemolysin chain II precursor | 011_1955 | 046_0267 | 0,4525 | 0,1215 |
| *hla £* | Alpha-hemolysin | 011_1514 | 046_1259 | 2,018 | 4,261 |
| **toxin genes** |  |  |  | - | - |
| *set10* | Exotoxin 7 | 011_0061 | 046_0944 | - | 2,041 |
| *lukE* | Leukotoxin LukE | 011_0686 | 046_2483 | 0,33066667 | - |
| *lukD* | Leukotoxin LukD | 011_0685 | 046_2484 | 0,487 | - |
| --- | Exotoxin 6 | 011_0062 | 046_0945 | 2,04 | - |
| --- | Toxin beta-grasp domain protein | 011_0809 | 046_1931 | 2,256 | - |
| --- | Toxin beta-grasp domain protein | 011_0811 | 046_1929 | 2,59325 | - |
| --- | Toxin beta-grasp domain-containing protein | 011_0810 | 046_1930 | 2,68433333 | - |
| *set8* | Exotoxin 8 | 011_0060 | 046_0943 | 3,21 | 0,441 |

^1^: Genes are classified in GO functional classes.

^2^: Names are given according to annotation of available *S. aureus* sequence genomes.

^3^: Coding sequence numbers corresponding to genes in *S. aureus* O11 and S. aureus O46

^4^: Expression ratio between *S. aureus* O11 and *S. aureus* O46 (O11/O46) during log phase (Expo) and stationary phase (Stat) (Ratio higher than 2 indicate overexpression in O11 and lower than 0.5 indicate overexpression in O46)

£: Difference confirmed by proteomic analysis

*: Results confirmed by qRT-PCR
