# Supplementary material for: Molecular Basis of Virulence in Staphylococcus aureus Mastitis
Source: PLoS One. 2011 Nov 11;6(11):e27354. doi: 10.1371/journal.pone.0027354 (PMC3214034; doi:10.1371/journal.pone.0027354)
Supplement: Table S4 — Expression profiles of genes exhibiting significant variations between O11 and O46 during log phase in deferoxamine-RPMI medium. (DOC) [file pone.0027354.s004.doc]

**Table S4:** Expression profiles of genes exhibiting significant variations between O11 and O46 during log phase in deferoxamine-RPMI medium

| **SA number^1^** | **Gene^2^** | **mean foldchange^3^** | **Description^4^** | **O11 CDS^5^** | **O46CDS^5^** |
| --- | --- | --- | --- | --- | --- |
| **Energy production and conversion** |  |  |  |  |  |
| SA2406 | cudA | 0,45 | glycine betaine aldehyde dehydrogenase gbsA | 011_0124 | 046_0209 |
| SA1142 | glpD | 0,45 | aerobic glycerol-3-phosphate dehydrogenase | 011_0955 | 046_0801 |
| SA1863 | leuB | 0,45 | 3-isopropylmalate dehydrogenase | 011_2745 | 046_2645 |
| SA1184 | citB | 0,46 | aconitate hydratase | 011_1006 | 046_0851 |
| SA2183 | narJ | 2,01 | Nitrate reductase molybdenum cofactor assembly chaperone | 011_1928 | 046_0241 |
| SA0551 | --- | 2,35 | Mercuric reductase | 011_1575 | 046_1630 |
| SA0757 | --- | 2,36 | Nitroreductase | 011_2083 | 046_2515 |
| **Cell cycle control and mitosis** |  |  |  |  |  |
| SA0276 | --- | 2,09 | Protein essC | 011_1683 | 046_0651 |
| SA1077 | --- | 3,33 | chromosome segregation SMC protein | 011_0883 | 046_2589 |
| **Amino Acid metabolism and transport** |  |  |  |  |  |
| SA0242 | --- | 0,23 | Alcohol dehydrogenase GroES domain protein | 011_0009 | 046_0543 |
| SA2202 | --- | 0,28 | Extracellular solute-binding protein, family 3 | 011_1949 | 046_0261 |
| SA2200 | --- | 0,29 | ABC transporter, ATP-binding protein | 011_1947 | 046_0259 |
| SA2201 | --- | 0,29 | Amino acid ABC transproter, permease protein, putative | 011_1948 | 046_0260 |
| SA0303 | --- | 0,32 | Putative sodium/glucose cotransporter | 011_1651 | 046_0681 |
| SA2319 | --- | 0,35 | L-serine dehydratase, iron-sulfur-dependent, beta subunit | 011_0208 | 046_0125 |
| SA1229 | dapD | 0,37 | putative tetrahydrodipicolinate acetyltransferase | 011_2730 | 046_1041 |
| SA2405 | cudB | 0,38 | choline dehydrogenase | 011_0125 | 046_0208 |
| SA0294 | --- | 0,39 | Branched-chain amino acid transport system II carrier protein | 011_1661 | 046_0671 |
| SA0304 | --- | 0,39 | N-acetylneuraminate lyase | 011_1650 | 046_0682 |
| SA0541 | --- | 0,41 | amino acid transporter | 011_1586 | 046_1641 |
| SA1228 | dapB | 0,42 | dihydrodipicolinate reductase | 011_2729 | 046_1042 |
| SA0430 | gltB | 0,43 | glutamate synthase large subunit | 011_0700 | 046_1933 |
| SA0431 | gltD | 0,44 | glutamate synthase subunit beta | 011_0699 | 046_1932 |
| SA1860 | ilvH | 0,44 | acetolactate synthase 1 regulatory subunit | 011_2748 | 046_2648 |
| SA1225 | lysC | 0,44 | aspartate kinase | 011_2726 | 046_1045 |
| SA1227 | dapA | 0,46 | dihydrodipicolinate synthase | 011_2728 | 046_1043 |
| SA1154 | --- | 0,46 | Aromatic amino acid beta-eliminating lyase/threonine aldolase | 011_0973 | 046_0819 |
| SA1862 | leuA | 0,47 | 2-isopropylmalate synthase | 011_2746 | 046_2646 |
| SA1858 | --- | 0,48 | dihydroxy-acid dehydratase | 011_2750 | 046_2650 |
| SA0200 | --- | 0,48 | Binding-protein-dependent transport systems inner membrane component | 011_0742 | 046_0491 |
| SA2327 | --- | 0,48 | pyruvate oxidase | 011_0199 | 046_0134 |
| SA1226 | asd | 0,48 | aspartate semialdehyde dehydrogenase | 011_2727 | 046_1044 |
| SA1424 | aroE | 0,49 | shikimate 5-dehydrogenase | 011_2661 | 046_2269 |
| SA2472 | hisZ | 2,10 | ATP phosphoribosyltransferase regulatory subunit | 011_1129 | 046_1508 |
| SA2217 | --- | 2,13 | ABC transporter ATP-binding protein | 011_1966 | 046_0278 |
| SA2082 | ureA | 2,23 | urease subunit gamma | 011_0569 | 046_1147 |
| SACOL1477 | tdcB | 2,25 | threonine dehydratase | 011_2103 | 046_1703 |
| SA2468 | hisB | 2,51 | imidazoleglycerol-phosphate dehydratase | 011_1125 | 046_1512 |
| SA2084 | ureC | 2,71 | urease subunit alpha | 011_0571 | 046_1145 |
| SA2083 | ureB | 2,74 | urease subunit beta | 011_0570 | 046_1146 |
| SA1272 | ald | 2,75 | alanine dehydrogenase | 011_2104 | 046_1704 |
| SA1436 | --- | 2,77 | Urea amidolyase related protein | 011_2476 | 046_2281 |
| SACOL1916 | --- | 2,84 | amino acid ABC transporter, permease/substrate-binding protein | 011_2011 | 046_2459 |
| **Nucleotide metabolism and transport** |  |  |  |  |  |
| SA0131 | deoD1 | 0,28 | purine nucleoside phosphorylase | 011_2722 | 046_2737 |
| SA1043 | pyrB | 2,09 | aspartate carbamoyltransferase catalytic subunit | 011_0848 | 046_1893 |
| SA1044 | pyrC | 2,26 | dihydroorotase | 011_0849 | 046_1892 |
| SA1042 | pyrP | 2,75 | uracil permease | 011_0847 | 046_1894 |
| **Carbohydrate metabolism and transport** |  |  |  |  |  |
| SA2204 | --- | 0,18 | 2,3-bisphosphoglycerate-dependent phosphoglyc... | 011_1952 | 046_0264 |
| SA0307 | --- | 0,33 | N-acetylmannosamine-6-phosphate 2-epimerase | 011_1647 | 046_0685 |
| SA0298 | --- | 0,34 | Regulatory protein PfoR | 011_1656 | 046_0677 |
| SA2203 | --- | 0,36 | EmrB/QacA family drug resistance transporter | 011_1950 | 046_0262 |
| SA2320 | --- | 0,39 | Regulatory protein PfoR | 011_0207 | 046_0126 |
| SA1963 | fmtB | 0,42 | mannitol-1-phosphate 5-dehydrogenase | 011_1071 | 046_0556 |
| SA0134 | drm | 0,46 | phosphopentomutase | 011_0807 | 046_0698 |
| SA1962 | mtlF | 0,49 | PTS system, mannitol specific IIA component | 011_1069 | 046_0558 |
| SA1269 | --- | 2,04 | major facilitator transporter | 011_2350 | 046_2559 |
| SA0182 | --- | 2,05 | Indole-3-pyruvate decarboxylase | 011_0756 | 046_0478 |
| SA2326 | glcB | 2,29 | PTS system, glucose-specific IIABC component | 011_0200 | 046_0133 |
| SA1599 | --- | 2,48 | putative translaldolase | 011_1872 | 046_2451 |
| SA1198 | --- | 4,73 | Peptidase M42 family protein | 011_1023 | 046_0869 |
| **Coenzyme metabolism** |  |  |  |  |  |
| SA1492 | hemB | 2,09 | delta-aminolevulinic acid dehydratase | 011_1311 | 046_0609 |
| SA2212 | --- | 2,23 | 8-amino-7-oxononanoate synthase | 011_1961 | 046_0273 |
| SA1608 | --- | 2,49 | S-adenosylmethionine synthetase | 011_2514 | 046_2557 |
| SA2213 | bioB | 2,66 | biotin synthase | 011_1962 | 046_0274 |
| SA2215 | bioD | 3,54 | dethiobiotin synthetase | 011_1964 | 046_0276 |
| SA2214 | bioA | 3,60 | adenosylmethionine-8-amino-7-oxononanoate aminotransferase | 011_1963 | 046_0275 |
| **Lipid metabolism** |  |  |  |  |  |
| SA0241 | --- | 0,22 | 2-C-methyl-D-erythritol 4-phosphate cytidylyltransferase | 011_0010 | 046_0542 |
| SA2260 | fabG | 0,46 | short chain dehydrogenase | 011_2419 | 046_2097 |
| SA0415 | --- | 2,21 | Phosphoesterase PA-phosphatase related | 011_0716 | 046_1949 |
| SA2323 | --- | 2,24 | Alpha/beta hydrolase fold-3 domain protein | 011_0203 | 046_0130 |
| **Translation** |  |  |  |  |  |
| SA2034 | rpsH | 0,44 | 30S ribosomal protein S8 | 011_0517 | 046_1199 |
| SA0942 | def | 0,48 | peptide deformylase | 011_1439 | 046_2062 |
| **Transcription** |  |  |  |  |  |
| SA1805 | --- | 0,10 | Transcription regulator |  | 046_2321 |
| SA1149 | glnR | 0,41 | glutamine synthetase repressor | 011_0962 | 046_0808 |
| SA2358 | --- | 0,43 | TetR family regulatory protein | 011_0166 | 046_0168 |
| SA0235 | --- | 0,43 | BglG family transcriptional antiterminator | 011_0017 | 046_0535 |
| SA1999 | --- | 0,46 | NAD-dependent deacetylase | 011_1836 | 046_1695 |
| SA1984 | asp23 | 0,49 | alkaline shock protein 23 | 011_1851 | 046_1680 |
| SA0337 | --- | 2,05 | Cro/CI family transcriptional regulator | 011_2675 | 046_1564 |
| SA2115 | --- | 2,10 | RpiR family transcriptional regulator, glv operon transcriptional regulator | 011_0607 | 046_1109 |
| SA1351 | ahrC | 2,12 | arginine repressor | 011_2170 | 046_2156 |
| SA0552 | --- | 2,13 | Transcriptional regulator, BadM/Rrf2 family | 011_1574 | 046_1631 |
| SA1595 | --- | 2,69 | RNA polymerase sigma factor sigS | 011_1876 | 046_2447 |
| SA2308 | --- | 3,12 | Transcriptional regulator, MarR family | 011_0219 | 046_0114 |
| SA0882 | --- | 5,19 | Competence transcription factor ComK | 011_2624 | 046_0424 |
| **Replication and repair** |  |  |  |  |  |
| SAV0869 | --- | 0,05 | hypothetical protein |  | 046_2761 |
| SA0746 | nuc | 0,38 | staphylococcal nuclease | 011_2070 | 046_2528 |
| SA1328 | xerD | 2,21 | site-specific recombinase | 011_2310 | 046_1871 |
| SAR1541 | polA | 4,98 | putative DNA polymerase | 011_1630 | 046_2661 |
| SA1196 | --- | 20,37 | ImpB/MucB/SamB family protein | 011_1021 | 046_0866 |
| **Cell wall/membrane/envelop biogenesis** |  |  |  |  |  |
| SA0243 | --- | 0,37 | Teichoic acid biosynthesis protein B | 011_0008 | 046_0544 |
| SA1231 | alr | 0,39 | Alanine racemase 2 | 011_2732 | 046_1039 |
| SA2328 | cidB | 0,48 | Holin-like protein cidB | 011_0198 | 046_0135 |
| **Post-translational modification, protein turnover, chaperone functions** |  |  |  |  |  |
| SA2414 | --- | 0,44 | glutathione peroxidase | 011_0113 | 046_0219 |
| SA0723 | clpP | 0,46 | ATP-dependent Clp protease, proteolytic subunit ClpP | 011_2345 | 046_2232 |
| SA0815 | --- | 2,12 | Putative peptidyl-prolyl cis-trans isomerase | 011_0329 | 046_0351 |
| SA2085 | ureF | 2,15 | urease accessory protein UreE | 011_0573 | 046_1144 |
| SA1146 | --- | 2,35 | Glutathione peroxidase homolog bsaA | 011_0959 | 046_0805 |
| SA2336 | clpL | 2,85 | ATP-dependent Clp proteinase chain clpL | 011_0190 | 046_0143 |
| **Inorganic ion transport and metabolism** |  |  |  |  |  |
| SAR0261 | --- | 0,28 | putative nitric oxide reductase | 011_1707 | 046_0627 |
| SA2486 | --- | 0,43 | DASS family divalent anion:Na+ symporter | 011_1144 | 046_1493 |
| SA0422 | --- | 0,44 | NLPA lipoprotein | 011_0708 | 046_1941 |
| SA0132 | --- | 0,47 | Major facilitator superfamily MFS_1 | 011_2723 | 046_2738 |
| SA0421 | glcA | 0,47 | ABC transporter, permease protein | 011_0754 | 046_0480 |
| SA1912 | atpI | 0,49 | ATP synthase I | 011_2057 | 046_0905 |
| SAR0461 | --- | 0,50 | Methionine import ATP-binding protein metN 1 | 011_0710 | 046_1943 |
| SA0100 | --- | 2,52 | Na/Pi cotransporter family protein | 011_1432 | 046_1413 |
| SA2322 | --- | 3,26 | DMT superfamily drug/metabolite transporter | 011_0204 | 046_0129 |
| **iron metabolism genes** |  |  |  |  |  |
| SA0114 | sbnC | 2,02 | Siderophore staphylobactin biosynthesis protein | 011_1342 | 046_1402 |
| SA0109 | sirC | 3,15 | Iron-regulated ABC transporter siderophore permease protein SirC | 011_1347 | 046_1407 |
| SA0110 | sirB | 3,34 | Iron-regulated ABC transporter siderophore permease protein SirB | 011_1346 | 046_1406 |
| SA0111 | sirA | 3,92 | Iron-regulated ABC transporter siderophore-binding protein SirA | 011_1345 | 046_1405 |
| SA1552 | isdH | 7,29 | Iron-regulated surface determinant protein H | 011_1248 | 046_1353 |
| **Secondary Structure** |  |  |  |  |  |
| SA0805 | --- | 2,07 | Thioesterase | 011_0339 | 046_0341 |
| SA0181 | --- | 2,07 | Isochorismatase hydrolase | 011_0757 | 046_0477 |
| **General Functional Prediction only** |  |  |  |  |  |
| SAV1989 | --- | 0,01 | Putative aryl-alcohol dehydrogenase |  | 046_2787 |
| SA1757 | --- | 0,14 | truncated amidase |  | 046_1942 |
| SA1536 | --- | 0,18 | hypothetical protein | 011_1265 | 046_1370 |
| SA1321 | --- | 0,20 | Lipoprotein, putative |  | 046_2733 |
| SA1319 | --- | 0,26 | putative lipoprotein |  | 046_2734 |
| SA1230 | --- | 0,36 | hippurate hydrolase | 011_2731 | 046_1040 |
| SA0724 | --- | 0,38 | Epimerase family protein | 011_2343 | 046_2234 |
| SA0840 | --- | 0,39 | PEBP family protein | 011_0304 | 046_0379 |
| SA2158 | --- | 0,44 | Lipoprotein | 011_0656 | 046_1060 |
| SA2403 | --- | 0,44 | Antibiotic biosynthesis monooxygenase | 011_0127 | 046_0206 |
| SAB1348c | --- | 0,45 | Lipoprotein | 011_2320 | 046_1862 |
| SA1990 | --- | 0,47 | Putative cell surface hydrolase | 011_1845 | 046_1686 |
| SACOL0270 | --- | 0,48 | staphyloxanthin biosynthesis protein, putative | 011_1689 | 046_0645 |
| SA2448 | --- | 0,50 | FMN-binding flavin reductase family protein | 011_1101 | 046_1535 |
| SA2155 | mqo | 0,50 | malate:quinone oxidoreductase | 011_0653 | 046_1063 |
| SAR0841 | --- | 2,03 | putative acetyltransferase | 011_2327 | 046_2250 |
| SA2173 | --- | 2,06 | Putative exported protein | 011_1920 | 046_0233 |
| SAS025 | --- | 2,23 | Putative membrane protein | 011_0302 | 046_0381 |
| MW1408 | --- | 2,23 | Virulence-associated protein E | 011_2618 | 046_2681 |
| SA1002 | --- | 2,26 | Putative membrane protein | 011_1508 | 046_1265 |
| SA0185 | murQ | 2,40 | N-acetylmuramic acid-6-phosphate etherase | 011_0752 | 046_0482 |
| SA1739 | --- | 2,41 | Peptidase C45 | 011_1735 | 046_2379 |
| SA2267 | --- | 2,68 | IraB protein | 011_0255 | 046_2603 |
| SA0368 | --- | 8,79 | Sodium:dicarboxylate symporter | 011_0083 | 046_0966 |
| **defense/virulence factor** |  |  |  |  |  |
| SA0841 | --- | 3,13 | MAP domain-containing protein | 011_0303 | 046_0380 |
| **capsule genes** |  |  |  |  |  |
| SAR0158 | cap8H | 0,30 | capsular polysaccharide synthesis enzyme capH | 011_0788 | 046_0717 |
| SA0147 | capD | 0,37 | capsular polysaccharide synthesis enzyme CapD | 011_0792 | 046_0713 |
| SAR0161 | cap8K | 0,37 | capsular polysaccharide synthesis enzyme capK | 011_0785 | 046_0721 |
| SA0156 | capM | 0,40 | capsular polysaccharide synthesis enzyme CapM | 011_0782 | 046_0723 |
| SA0155 | capL | 0,40 | capsular polysaccharide synthesis enzyme CapL | 011_0783 | 046_0722 |
| SA0157 | capN | 0,42 | capsular polysaccharide synthesis enzyme CapN | 011_0781 | 046_0724 |
| SA0159 | capP | 0,42 | capsular polysaccharide synthesis enzyme CapP | 011_0779 | 046_0726 |
| SAR0159 | cap8I | 0,43 | capsular polysaccharide synthesis enzyme capI | 011_0787 | 046_0718 |
| SA0149 | capF | 0,45 | capsular polysaccharide synthesis enzyme CapF | 011_0790 | 046_0715 |
| SA0148 | capE | 0,47 | capsular polysaccharide synthesis enzyme CapE | 011_0791 | 046_0714 |
| SA0150 | capG | 0,48 | capsular polysaccharide synthesis enzyme CapG | 011_0789 | 046_0716 |
| SA0158 | capO | 0,48 | capsular polysaccharide synthesis enzyme CapO | 011_0780 | 046_0725 |
| **adhesion genes** |  |  |  |  |  |
| SA0742 | clfA | 0,21 | Clumping factor A | 011_2325 | 046_2251 |
| SA2290 | fnbB | 0,29 | FnbB protein |  | 046_2117 |
| SA0521 | sdrE | 2,18 | Serine-aspartate repeat-containing protein E | 011_2763 | 046_2767 |
| SA0520 | sdrD | 5,11 | Serine-aspartate repeat-containing protein D | 011_2683 |  |
| **enzyme genes** |  |  |  |  |  |
| SA1628 | splD | 0,42 | serine protease SplD | 011_0672 | 046_2496 |
| SA1726 | --- | 2,34 | Staphostatin A superfamily | 011_1719 | 046_2363 |
| SA1725 | scpA | 3,29 | Staphopain A | 011_1718 | 046_2362 |
| SAR1902 | splE | 155,49 | Serine protease splE | 011_0673 |  |
| **hemolysin genes** |  |  |  |  |  |
| SA2207 | hlgA | 0,45 | gamma-hemolysin chain II precursor | 011_1955 | 046_0267 |
| SA1007 | hla | 2,02 | alpha-hemolysin | 011_1514 | 046_1259 |
| **toxin genes** |  |  |  |  |  |
| SA1638 | lukE | 0,33 | leukotoxin LukE | 011_0686 | 046_2483 |
| SA1637 | lukD | 0,49 | leukotoxin LukD | 011_0685 | 046_2484 |
| SA0382 | --- | 2,04 | Exotoxin 6 | 011_0062 | 046_0945 |
| SA1009 | --- | 2,26 | Toxin beta-grasp domain protein | 011_0809 | 046_1931 |
| SA1011 | --- | 2,59 | Toxin beta-grasp domain protein | 011_0811 | 046_1929 |
| SA1010 | --- | 2,68 | Toxin beta-grasp domain-containing protein | 011_0810 | 046_1930 |
| SA0384 | set8 | 3,21 | Exotoxin 8 | 011_0060 | 046_0943 |
| **stress response** |  |  |  |  |  |
| SA2324 | --- | 2,07 | Thioredoxin | 011_0202 | 046_0131 |
| **Function Unknown** |  |  |  |  |  |
| SA1798 | --- | 0,02 | hypothetical protein |  | 046_2787 |
| SA2359 | --- | 0,03 | hypothetical protein | 011_0165 | 046_0169 |
| SA2360 | --- | 0,04 | hypothetical protein | 011_0164 | 046_0170 |
| SA1320 | --- | 0,12 | hypothetical protein |  | 046_2732 |
| MW1542 | --- | 0,28 | hypothetical protein | 011_2656 | 046_2264 |
| MW1419 | --- | 0,29 | hypothetical protein | 011_1183 | 046_2730 |
| SA0883 | --- | 0,35 | Uncharacterised conserved protein UCP007165 | 011_2625 | 046_0425 |
| SAS049 | --- | 0,38 | hypothetical protein | 011_2459 | 046_2298 |
| SA0712 | --- | 0,39 | Putative membrane protein | 011_2587 | 046_2221 |
| SA0141 | --- | 0,44 | hypothetical protein | 011_0799 | 046_0706 |
| SA2262 | --- | 0,45 | Alkylhydroperoxidase AhpD family protein | 011_2435 | 046_2112 |
| SAS053 | --- | 0,48 | hypothetical protein | 011_2016 | 046_2464 |
| SA1376 | --- | 0,48 | hypothetical protein | 011_2196 | 046_2182 |
| SAB0370 | --- | 0,49 | hypothetical protein | 011_0070 | 046_0953 |
| SA0269 | --- | 2,02 | hypothetical protein | 011_1690 | 046_0644 |
| SA0749 | --- | 2,02 | hypothetical protein | 011_2073 | 046_2525 |
| SAS068 | --- | 2,21 | hypothetical protein | 011_2371 | 046_1824 |
| SA1153 | --- | 2,23 | hypothetical protein | 011_0968 | 046_0814 |
| SAS088 | --- | 2,25 | hypothetical protein | 011_0189 | 046_0144 |
| SA0542 | --- | 2,49 | Putative membrane protein | 011_1585 | 046_1640 |
| SAV0787 | --- | 2,58 | hypothetical protein | 011_1548 | 046_1001 |
| SA1258 | --- | 2,68 | UPF0230 | 011_2600 | 046_1851 |
| SA0543 | --- | 2,93 | Hypothetical membrane protein | 011_1584 | 046_1639 |
| SAOUHSC_A01912 | --- | 3,15 | hypothetical protein | 011_2740 |  |
| SA0290 | --- | 3,23 | hypothetical protein | 011_1665 |  |
| SA0086 | --- | 3,47 | hypothetical protein | 011_1404 | 046_1439 |
| SA0830 | --- | 3,59 | hypothetical protein | 011_0313 | 046_0368 |
| SAR0401a | --- | 4,15 | hypothetical protein | 011_0082 | 046_0965 |
| SA1787 | --- | 4,59 | hypothetical protein | 011_1627 | 046_2658 |
| SA0748 | --- | 5,19 | hypothetical protein | 011_2072 | 046_2526 |
| SA1807 | --- | 30,63 | hypothetical protein | 011_0461 |  |
| **mobile genome elements** |  |  |  |  |  |
| **Pathogenicity island** |  |  |  |  |  |
| SA1827 | --- | 2,43 | Pathogenicity island protein | 011_0422 | 046_0996 |
| SA1821 | --- | 2,66 | mobile element-associated protein (pid:82751613) |  | 046_0990 |
| SA1830 | --- | 2,48 | Pathogenicity island protein | 011_1550 | 046_0999 |
| SAR0374 | --- | 2,90 | pathogenicity island protein (pid:82750111) | 011_0424 | 046_0998 |
| SA1738 | --- | 4,39 | YolD-like protein | 011_1733 | 046_2377 |
| SAV0789 | --- | 5,29 | mobile element-associated protein (pid:82751624) | 011_2685 |  |
| SACOL0892 | --- | 8,02 | Pathogenicity island protein | 011_2689 | 046_1002 |
| SAB0348 | --- | 18,41 | Pathogenicity island protein Orf17 | 011_2686 |  |
| **Phage** |  |  |  |  |  |
| SAB1756c | --- | 0,00 | phage-like protein |  | 046_2440 |
| SAUSA300_1427 | --- | 0,00 | phiSLT ORF86-like protein |  | 046_2788 |
| SAB1752c | --- | 0,00 | hypothetical protein |  | 046_2785 |
| SAV0870 | --- | 0,01 | hypothetical protein |  | 046_2761 |
| SAOUHSC_02086 | --- | 0,01 | PV83 orf 4-like protein-related protein |  | 046_2323 |
| SACOL0343 | --- | 0,02 | prophage L54a, replicative DNA helicase, putative |  | 046_2794 |
| SACOL0338 | --- | 0,02 | Phage protein |  | 046_2790 |
| SAB1760 | --- | 0,02 | integrase |  | 046_2435 |
| SAV0862 | --- | 0,02 | Virulence-related phage protein |  | 046_2789 |
| SACOL0345 | --- | 0,03 | Conserved hypothetical phage protein |  | 046_2844 |
| SACOL0341 | --- | 0,03 | hypothetical protein | 011_0479 | 046_2760 |
| SACOL0339 | --- | 0,03 | prophage L54a, single-stranded DNA binding protein | 011_0477 | 046_2840 |
| SAV0851 | --- | 0,04 | Putative phage regulatory protein |  | 046_0694 |
| SAV1979 | --- | 0,05 | phi PVL ORF 50-like protein | 011_0486 | 046_2671 |
| SAB1742c | --- | 0,05 | Gp18 |  | 046_2795 |
| SAV0879 | --- | 0,05 | Phage77_ORF072 protein |  | 046_2689 |
| SACOL0348 | --- | 0,07 | hypothetical protein | 011_0484 | 046_2801 |
| SAOUHSC_02058 | --- | 0,08 | Conserved hypothetical phage protein |  | 046_2027 |
| SAV0882 | --- | 0,08 | int gene activator RinB |  | 046_2691 |
| SAV1998 | --- | 0,12 | repressor-like protein |  | 046_2321 |
| SACOL0344 | --- | 0,13 | hypothetical protein |  | 046_2793 |
| SA1784 | --- | 0,17 | DUTPase | 011_2612 | 046_2729 |
| SAS1881 | --- | 0,25 | hypothetical protein | 011_1203 | 046_2007 |
| SA0214 | --- | 0,26 | sugar phosphate antiporter | 011_0045 | 046_0507 |
| SAV0866 | --- | 0,28 | phage-like protein (pid:82751465) | 011_0478 |  |
| SAB1748c | --- | 0,34 | phage-like protein | 011_0474 | 046_2771 |
| SA1786 | --- | 0,37 | Phage conserved open reading frame 51 | 011_0487 | 046_2835 |
| SA1785 | --- | 0,38 | Phi PVL orf 52-like protein | 011_1183 | 046_2730 |
| SAR1536 | --- | 0,39 | Hypothetical phage protein | 011_1626 |  |
| SAR1552 | --- | 0,39 | Hypothetical phage protein |  | 046_2669 |
| SA1782 | --- | 0,39 | phage-like protein (pid:82751451) | 011_1185 | 046_2688 |
| SAV1977 | --- | 0,42 | phi PV83 orf 27-like protein | 011_2758 | 046_2798 |
| SACOL0352 | --- | 0,43 | hypothetical protein orf29 | 011_2757 |  |
| SAOUHSC_02077 | --- | 0,45 | phi PV83 orf 12-like protein-related protein | 011_0472 | 046_2773 |
| SAOUHSC_02089 | --- | 0,47 | phage family integrase | 011_0459 | 046_2325 |
| SAOUHSC_02203 | --- | 0,48 | Conserved hypothetical phage protein | 011_2615 | 046_2678 |
| SACOL0389 | --- | 0,50 | prophage L54a, amidase, putative | 011_1802 | 046_1595 |
| SACOL0375 | --- | 2,14 | prophage L54a, major tail protein, putative | 011_1818 | 046_1611 |
| SAOUHSC_02217 | --- | 2,16 | phi ETA orf 22-like protein | 011_0480 |  |
| SACOL0385 | --- | 2,16 | PhiSLT ORF129-like protein | 011_1807 | 046_1600 |
| SACOL0318 | --- | 2,28 | prophage L54a, integrase | 011_1644 | 046_0689 |
| SAS1882 | --- | 2,36 | Hypothetical phage protein | 011_1202 | 046_2008 |
| SACOL0362 | --- | 2,44 | Putative exported phage protein | 011_2617 | 046_2680 |
| SACOL0376 | --- | 2,51 | prophage L54a, major tail protein, putative | 011_1816 | 046_1609 |
| MW1402 | --- | 2,53 | phiSLT ORF 101-like protein, terminase, small subunit (pid:87161237) | 011_1828 | 046_1620 |
| SAUSA300_1436 | --- | 2,82 | phiSLT ORF144-like protein, putative lipoprotein | 011_1641 | 046_0692 |
| SACOL0334 | --- | 3,46 | Hypothetical phage protein | 011_1635 | 046_2667 |
| SACOL0333 | --- | 3,66 | Putative DNA-binding protein | 011_1636 | 046_2668 |
| SAR1540 | --- | 5,14 | Hypothetical phage protein | 011_1629 | 046_2660 |
| SAR1542 | --- | 5,53 | Hypothetical phage protein | 011_1631 | 046_2663 |
| SAS0911 | --- | 5,58 | phiSLT ORF122-like protein, DNA polymerase (pid:87160354) | 011_1628 | 046_2659 |
| SAB1736c | --- | 5,80 | phage-like protein | 011_1627 | 046_2658 |
| SACOL0889 | --- | 6,19 | phiSLT ORF153-like protein (pid:87161835) | 011_0464 | 046_0693 |
| SAR1514 | --- | 6,43 | Hypothetical phage protein | 011_1821 | 046_1614 |
| SAR1544 | --- | 8,23 | Hypothetical phage protein | 011_1633 | 046_2665 |
| SAR1543 | --- | 12,21 | Hypothetical phage protein | 011_1632 | 046_2664 |
| SAR1545 | --- | 12,34 | Hypothetical phage protein | 011_1634 | 046_2666 |
| SAR2094 | --- | 20,31 | Hypothetical phage protein | 011_1638 |  |
| SAOUHSC_02206 | --- | 24,22 | Hypothetical phage protein | 011_2611 | 046_1902 |
| SACOL0885 | --- | 173,00 | pathogenicity island protein, integrase | 011_2694 |  |

^1^: Genes are classified in GO functional classes and coding sequence number corresponding to the closer available sequenced strain is indicated

^2^: *S. aureus* gene names

^3^: Expression ratio between *S. aureus* O11 and *S. aureus* O46 during log phase (Ratio higher than 2 indicate overexpression in O11 and lower than 0.5 indicate overexpression in O46)

^4^: Names are given according to annotation of available *S. aureus* sequence genomes.

^5^: Coding sequence numbers corresponding to the identified proteins in *S. aureus* O11 and *S. aureus* O46
