# Supplementary material for: Molecular Basis of Virulence in Staphylococcus aureus Mastitis
Source: PLoS One. 2011 Nov 11;6(11):e27354. doi: 10.1371/journal.pone.0027354 (PMC3214034; doi:10.1371/journal.pone.0027354)
Supplement: Table S5 — Expression profiles of genes exhibiting significant variations between O11 and O46 during stationary phase in deferoxamine-RPMI medium. (DOC) [file pone.0027354.s005.doc]

**Table S5:** Expression profiles of genes exhibiting significant variations between O11 and O46 during stationary phase in deferoxamine-RPMI medium

| **SA number^1^** | **Gene^2^** | **mean foldchange^3^** | **Description^4^** | **O11 CDS^5^** | **O46CDS^5^** |
| --- | --- | --- | --- | --- | --- |
| **Energy production and conversion** |  |  |  |  |  |
| SA2406 | cudA | 0,46 | glycine betaine aldehyde dehydrogenase gbsA | 011_0124 | 046_0209 |
| SA1262 | cvfC | 2,04 | Conserved virulence factor C | 011_2604 | 046_1855 |
| SA2131 | --- | 2,10 | Sodium transport system permease | 011_0626 | 046_1090 |
| SA1184 | citB | 2,37 | aconitate hydratase | 011_1006 | 046_0851 |
| SA0757 | --- | 3,36 | Nitroreductase | 011_2083 | 046_2515 |
| SAR0600 | --- | 5,78 | pyridine nucleotide-disulphide oxidoreductase protein | 011_1575 | 046_1630 |
| SAB0723 | --- | 5,23 | malate dehydrogenase | 011_2344 | 046_2233 |
| **Cell cycle control and mitosis** |  |  |  |  |  |
| SA0276 | --- | 0,40 | Protein essC | 011_1683 | 046_0651 |
| SA1267 | --- | 2,04 | Extracellular matrix-binding protein | 011_2352 | 046_2561 |
| SA1023 | --- | 2,31 | cell division protein | 011_0826 | 046_1915 |
| **Amino Acid metabolism and transport** |  |  |  |  |  |
| SA2319 | --- | 0,27 | L-serine dehydratase, iron-sulfur-dependent, beta subunit | 011_0208 | 046_0125 |
| SA0304 | --- | 0,29 | N-acetylneuraminate lyase | 011_1650 | 046_0682 |
| SA0303 | --- | 0,33 | Putative sodium/glucose cotransporter | 011_1651 | 046_0681 |
| SA1214 | --- | 0,38 | oligopeptide transporter permease | 011_1040 | 046_0887 |
| SA0242 | --- | 0,41 | Alcohol dehydrogenase GroES domain protein | 011_0009 | 046_0543 |
| SA2202 | --- | 0,45 | Extracellular solute-binding protein, family 3 | 011_1949 | 046_0261 |
| SA2405 | cudB | 0,45 | choline dehydrogenase | 011_0125 | 046_0208 |
| SA1216 | --- | 0,50 | Oligoendopeptidase F | 011_1042 | 046_0889 |
| SAB0056 | --- | 2,00 | ornithine cyclodeaminase protein | 011_1343 | 046_1403 |
| SA2076 | --- | 2,01 | GCN5-related N-acetyltransferase | 011_0563 | 046_1153 |
| SA2468 | hisB | 2,01 | imidazoleglycerol-phosphate dehydratase | 011_1125 | 046_1512 |
| SA1865 | leuD | 2,03 | isopropylmalate isomerase small subunit | 011_2743 | 046_2643 |
| SACOL0186 | --- | 2,03 | peptide ABC transporter, permease protein | 011_0742 | 046_0491 |
| SA1166 | thrB | 2,05 | homoserine kinase | 011_0987 | 046_0832 |
| SA1862 | leuA | 2,07 | 2-isopropylmalate synthase | 011_2746 | 046_2646 |
| SA0418 | --- | 2,08 | pyridoxal-phosphate dependent enzyme | 011_0712 | 046_1945 |
| SA1866 | ilvA | 2,10 | threonine dehydratase | 011_2742 | 046_2642 |
| SA0112 | --- | 2,16 | Cysteine synthase | 011_1344 | 046_1404 |
| SACOL1406 | trpC | 2,16 | indole-3-glycerol-phosphate synthase | 011_1027 | 046_0873 |
| SA2470 | hisD | 2,21 | histidinol dehydrogenase | 011_1127 | 046_1510 |
| SA2084 | ureC | 2,25 | urease subunit alpha | 011_0571 | 046_1145 |
| SAR1178 | pyrAA | 2,28 | carbamoyl phosphate synthase small subunit | 011_0850 | 046_1891 |
| SA1213 | --- | 2,33 | putative oligopeptide transport system permease | 011_1039 | 046_0885 |
| SA1232 | lysA | 2,33 | diaminopimelate decarboxylase | 011_2733 | 046_1038 |
| SA1203 | trpF | 2,34 | N-(5'-phosphoribosyl)anthranilate isomerase | 011_1028 | 046_0874 |
| SACOL1916 | --- | 2,39 | amino acid ABC transporter, permease/substrate-binding protein | 011_2011 | 046_2459 |
| SA1980 | --- | 2,53 | Alanine racemase domain protein | 011_1855 | 046_1676 |
| SA2083 | ureB | 2,76 | urease subunit beta | 011_0570 | 046_1146 |
| SA1150 | glnA | 2,82 | glutamine-ammonia ligase | 011_0963 | 046_0809 |
| SACOL1414 | --- | 2,89 | peptide ABC transporter, ATP-binding protein | 011_1037 | 046_0883 |
| SA2190 | --- | 3,13 | acetyltransferase (GNAT) family protein | 011_1935 | 046_0249 |
| SA1197 | --- | 3,46 | prephenate dehydrogenase | 011_1022 | 046_0867 |
| **Nucleotide metabolism and transport** |  |  |  |  |  |
| SA1938 | pyn | 2,16 | pyrimidine-nucleoside phosphorylase | 011_2030 | 046_0932 |
| SA1043 | pyrB | 2,30 | aspartate carbamoyltransferase catalytic subunit | 011_0848 | 046_1893 |
| SA0016 | purA | 2,80 | adenylosuccinate synthetase | 011_1182 | 046_1455 |
| SA1044 | pyrC | 2,80 | dihydroorotase | 011_0849 | 046_1892 |
| SA1042 | pyrP | 3,86 | uracil permease | 011_0847 | 046_1894 |
| SA1041 | pyrR | 2,52 | bifunctional pyrimidine regulatory protein PyrR uracil phosphoribosyltransferase | 011_0846 | 046_1895 |
| **Carbohydrate metabolism and transport** |  |  |  |  |  |
| SAV1989 |  | 0,01 | Putative aryl-alcohol dehydrogenase |  | 046_2787 |
| SA1784 | --- | 0,10 | DUTPase | 011_2612 | 046_2026 |
| SA0214 | uhpT | 0,24 | sugar phosphate antiporter | 011_0045 | 046_0507 |
| SA0233 | --- | 0,37 | PTS system, glucose-like IIB subunint | 011_0019 | 046_0533 |
| SA0307 | --- | 0,38 | N-acetylmannosamine-6-phosphate 2-epimerase | 011_1647 | 046_0685 |
| SA1962 | mtlF | 0,42 | PTS system, mannitol-specific IIA component | 011_1069 | 046_0558 |
| SA2204 | --- | 0,45 | 2,3-bisphosphoglycerate-dependent phosphoglycerate mutase | 011_1952 | 046_0264 |
| SA2279 | --- | 0,45 | Phosphoglucomutase | 011_0242 | 046_2613 |
| SA0905 | atl | 0,49 | autolysin, N-acetylmuramyl-L-alanine amidase and endo-b-N-acetylglucosaminidas | 011_1991 | 046_0320 |
| SA2480 | drp35 | 0,50 | Drp35 | 011_1137 | 046_1500 |
| SA0958 | --- | 2,25 | Inositol-phosphate phosphatase | 011_1455 | 046_2078 |
| SA1269 | --- | 3,22 | major facilitator transporter | 011_2350 | 046_2559 |
| SA1599 | --- | 4,78 | putative translaldolase | 011_1872 | 046_2451 |
| SAS020 | --- | 4,79 | Phosphoglycerate mutase | 011_2078 | 046_2520 |
| SA1198 | --- | 7,07 | Peptidase M42 family protein | 011_1023 | 046_0869 |
| SA2326 | glcB | 9,94 | PTS system, glucose-specific IIABC component | 011_0200 | 046_0133 |
| SA2320 | --- | 0,35 | Regulatory protein PfoR | 011_1656 | 046_0677 |
| **Coenzyme metabolism** |  |  |  |  |  |
| SA1896 | thiD | 2,03 | phosphomethylpyrimidine kinase | 011_2362 | 046_1833 |
| SA2215 | bioD | 2,04 | dethiobiotin synthetase | 011_1964 | 046_0276 |
| SA1494 | hemC | 2,05 | porphobilinogen deaminase | 011_1309 | 046_0607 |
| SA1493 | hemD | 2,14 | uroporphyrinogen III synthase | 011_1310 | 046_0608 |
| SA2214 | bioA | 3,30 | adenosylmethionine-8-amino-7-oxononanoate aminotransferase | 011_1963 | 046_0275 |
| SA1608 | --- | 4,06 | S-adenosylmethionine synthetase | 011_2514 | 046_2557 |
| **Lipid metabolism** |  |  |  |  |  |
| SA0241 | --- | 0,30 | 2-C-methyl-D-erythritol 4-phosphate cytidylyltransferase | 011_0010 | 046_0542 |
| SA2323 | --- | 2,04 | Alpha/beta hydrolase fold-3 domain protein | 011_0203 | 046_0130 |
| **Translation** |  |  |  |  |  |
| SA0754 | --- | 0,41 | Acetyltransferase, GNAT family | 011_2080 | 046_2518 |
| SA1456 | aspS | 2,06 | aspartyl-tRNA synthetase | 011_2454 | 046_2303 |
| SA1922 | rpmE2 | 2,60 | 50S ribosomal protein L31 type B | 011_2047 | 046_0915 |
| SA1040 | --- | 2,77 | Pseudouridine synthase | 011_0845 | 046_1896 |
| **Transcription** |  |  |  |  |  |
| SA0306 | --- | 0,31 | transcriptional activator of the glutamate synthase operon (LysR family) | 011_1648 | 046_0684 |
| SA1947 | czrA | 0,44 | repressor protein | 011_1058 | 046_0570 |
| SA0882 | --- | 0,46 | Competence transcription factor ComK | 011_2624 | 046_0424 |
| SA2358 | --- | 0,49 | TetR family regulatory protein | 011_0166 | 046_0168 |
| SA1872 | rsbU | 2,01 | sigmaB regulation protein RsbU | 011_2389 | 046_1806 |
| SA1516 | phoP | 2,22 | alkaline phosphatase synthesis transcriptional regulatory protein | 011_1287 | 046_0585 |
| SA0187 | --- | 2,23 | RpiR family transcriptional regulator | 011_0750 | 046_0484 |
| SA2062 | --- | 2,66 | HTH-type transcriptional regulator sarV | 011_0548 | 046_1168 |
| **Replication and repair** |  |  |  |  |  |
| SAV0869 |  | 0,03 | hypothetical protein |  | 046_2761 |
| SA1328 | xerD | 4,20 | Tyrosine recombinase xerD | 011_2310 | 046_1871 |
| SA1196 | --- | 9,90 | ImpB/MucB/SamB family protein | 011_1021 | 046_0866 |
| **Cell wall/membrane/envelop biogenesis** |  |  |  |  |  |
| SA0243 | --- | 0,47 | Teichoic acid biosynthesis protein B | 011_0008 | 046_0544 |
| SA2288 | gtaB | 0,47 | UTP-glucose-1-phosphate uridyltransferase | 011_0238 | 046_2617 |
| SA1022 | --- | 2,20 | S-adenosyl-L-methionine-dependent methyltransferase mraW | 011_0825 | 046_1916 |
| SA0710 | --- | 2,21 | LysM domain-containing protein | 011_2589 | 046_2219 |
| SA1024 | pbpA | 2,27 | penicillin-binding protein 1 | 011_0827 | 046_1914 |
| SA1959 | glmS | 2,39 | glucosamine--fructose-6-phosphate aminotransferase | 011_1066 | 046_0561 |
| SA1183 | opuD | 2,45 | glycine betaine transporter | 011_1005 | 046_0850 |
| SA0129 | --- | 2,50 | Surface protein SasD | 011_1325 | 046_1386 |
| SA2459 | icaA | 3,13 | N-glycosyltransferase PgaC | 011_1113 | 046_1523 |
| SA1616 | --- | 3,21 | Lipoprotein | 011_2522 | 046_2549 |
| **Cell motility** |  |  |  |  |  |
| SA1373 | --- | 2,02 | DNA transport machinery protein *ComGB* | 011_2193 | 046_2179 |
| **Post-translational modification, protein turnover, chaperone functions** |  |  |  |  |  |
| SA0723 | clpP | 0,50 | ATP-dependent Clp protease, proteolytic subunit ClpP | 011_2345 | 046_2232 |
| SA1495 | --- | 2,02 | hemA concentration negative effector hemX | 011_1308 | 046_0606 |
| SA2085 | ureE | 2,05 | urease accessory protein UreE | 011_0572 | 046_1144 |
| SA0815 | --- | 2,14 | Putative peptidyl-prolyl cis-trans isomerase | 011_0329 | 046_0351 |
| SA0365 | ahpF | 2,38 | alkyl hydroperoxide reductase subunit F | 011_0086 | 046_0969 |
| **Inorganic ion transport and metabolism** |  |  |  |  |  |
| SA2486 | --- | 0,08 | DASS family divalent anion:Na+ symporter | 011_1144 | 046_1493 |
| SAR0261 | --- | 0,19 | putative nitric oxide reductase | 011_1707 | 046_0627 |
| SA0263 | --- | 0,48 | Major facilitator superfamily MFS_1 | 011_1696 | 046_0638 |
| SA0690 | --- | 0,49 | ABC transporter ATP-binding protein | 011_2502 | 046_1236 |
| SA1941 | --- | 2,03 | general stress protein 20U | 011_1052 | 046_0576 |
| SAR2267 | --- | 2,04 | FecCD transport family protein | 011_1858 | 046_1673 |
| SA0619 | --- | 2,10 | Phosphate transporter family protein | 011_2240 | 046_0028 |
| SA1432 | --- | 2,28 | Putative membrane protein 5 | 011_2481 | 046_2277 |
| SA2420 | phoB | 2,31 | alkaline phosphatase III precursor | 011_0105 | 046_0227 |
| SA0807 | mnhG | 2,48 | putative monovalent cation/H+ antiporter subunit G | 011_0337 | 046_0343 |
| SA0578 | --- | 2,66 | putative monovalent cation/H+ antiporter subunit A | 011_2282 | 046_0070 |
| SA1148 | --- | 2,93 | Aluminium resistance family protein | 011_0961 | 046_0807 |
| SA0579 | --- | 2,94 | putative monovalent cation/H+ antiporter subunit B | 011_2281 | 046_0069 |
| SA0580 | --- | 3,31 | putative monovalent cation/H+ antiporter subunit C | 011_2280 | 046_0068 |
| SA0808 | mnhF | 3,58 | putative monovalent cation/H+ antiporter subunit F | 011_0336 | 046_0344 |
| SA1220 | pstC | 3,61 | Phosphate ABC transporter, permease PstC | 011_1046 | 046_0893 |
| SA1217 | PhoU | 3,68 | Phosphate transport system protein PhoU, putative | 011_1043 | 046_0890 |
| SA1219 | pstA | 5,44 | Phosphate ABC transporter, permease protein | 011_1045 | 046_0892 |
| SA1218 | pstB | 5,81 | phosphate ABC transporter, ATP-binding protein | 011_1044 | 046_0891 |
| SA0100 | --- | 6,49 | Na/Pi cotransporter family protein | 011_1432 | 046_1413 |
| SA1221 | pstS | 8,25 | Phosphate-binding protein pstS | 011_1047 | 046_0894 |
| **iron metabolism genes** |  |  |  |  |  |
| SA0160 | isdi | 0,10 | heme-degrading monooxygenase IsdI | 011_0778 | 046_0727 |
| SA0976 | isdB | 0,47 | Iron-regulated surface determinant protein B | 011_1476 | 046_1296 |
| SA0980 | isdE | 2,05 | High-affinity heme uptake system protein isdE | 011_1481 | 046_1291 |
| SA1315 | fer | 2,03 | ferredoxin | 011_2322 | 046_1751 |
| SA0688 | sstA | 2,31 | FecCD transport family protein 1 | 011_2504 | 046_1234 |
| SA0114 | sbnC | 2,68 | Siderophore staphylobactin biosynthesis protein | 011_1342 | 046_1402 |
| SA0110 | sirB | 3,21 | Iron-regulated ABC transporter siderophore permease protein SirB | 011_1346 | 046_1406 |
| SA1552 | isdH | 4,44 | Iron-regulated surface determinant protein H | 011_1248 | 046_1353 |
| SA0111 | sirA | 3,61 | Iron-regulated ABC transporter siderophore-binding protein SirA | 011_1345 | 046_1405 |
| SA0109 | sirC | 3,83 | Iron-regulated ABC transporter siderophore permease protein SirC | 011_1347 | 046_1407 |
| **Secondary Structure** |  |  |  |  |  |
| SAR0188 | --- | 2,08 | putative isochorismatase | 011_0757 | 046_0477 |
| **General Functional Prediction only** |  |  |  |  |  |
| SA1321 |  | 0,16 | Lipoprotein |  | 046_2733 |
| SA1319 |  | 0,31 | putative lipoprotein |  | 046_2734 |
| SA0840 | --- | 0,34 | PEBP family protein | 011_0304 | 046_0379 |
| SA0265 | lytM | 0,41 | peptidoglycan hydrolase | 011_1694 | 046_0640 |
| SA0309 | geh | 0,42 | glycerol ester hydrolase | 011_1645 | 046_0688 |
| SA0270 | --- | 0,42 | Staphyloxanthin biosynthesis protein | 011_1689 | 046_0645 |
| SA0423 | --- | 0,49 | N-acetylmuramoyl-L-alanine amidase | 011_0707 | 046_1940 |
| SA1209 | --- | 2,04 | hydrolase-related protein | 011_1035 | 046_0881 |
| SA2329 | cidA | 2,06 | LrgA family protein | 011_0197 | 046_0136 |
| SA1490 | --- | 2,11 | AbrB protein | 011_1314 | 046_0612 |
| SA2077 | --- | 2,21 | Biotin biosynthesis protein BioY | 011_0564 | 046_1152 |
| SA0185 | murQ | 2,27 | N-acetylmuramic acid-6-phosphate etherase | 011_0752 | 046_0482 |
| SA2306 | --- | 2,37 | Phospholipase/Carboxylesterase | 011_0221 | 046_0112 |
| SA0989 | --- | 2,60 | CvpA family protein | 011_1492 | 046_1280 |
| SA0753 | --- | 3,20 | Lysine exporter protein | 011_2079 | 046_2519 |
| SA0368 | --- | 11,87 | Sodium:dicarboxylate symporter | 011_0083 | 046_0966 |
| **Signal Transduction** |  |  |  |  |  |
| SA0536.1 | --- | 0,36 | Protein vraX | 011_1592 | 046_1647 |
| SA1515 | phoR | 2,51 | alkaline phosphatase synthesis sensor protein | 011_1288 | 046_0586 |
| **Intracellular trafficing and secretion** |  |  |  |  |  |
| SA2446 | --- | 2,29 | preprotein translocase subunit SecY | 011_1099 | 046_1537 |
| SA0826 | spsB | 2,85 | type-1 signal peptidase 1B | 011_0317 | 046_0364 |
| SA2445 | --- | 3,05 | accessory secretory protein Asp1 (pid:87162149) | 011_1098 | 046_1538 |
| **defense/virulence factor** |  |  |  |  |  |
| SA1750 | --- | 0,48 | truncated map-w protein | 011_1748 | 046_2392 |
| SA1751 | --- | 0,49 | Protein map | 011_1749 | 046_2393 |
| SA2217 | --- | 2,07 | ABC transporter ATP-binding protein 2 | 011_1966 | 046_0278 |
| SA0841 | --- | 2,38 | MAP domain-containing protein | 011_0303 | 046_0380 |
| SA2315 | --- | 2,42 | Antibiotic transport system permease | 011_0212 | 046_0121 |
| **capsule genes** |  |  |  |  |  |
| SACOL0140 | capE | 0,41 | capsular polysaccharide synthesis enzyme CapE | 011_0791 | 046_0714 |
| SA0150 | capG | 0,42 | capsular polysaccharide synthesis enzyme CapG | 011_0789 | 046_0716 |
| SACOL0138 | capC | 0,42 | capsular polysaccharide synthesis enzyme CapC | 011_0793 | 046_0712 |
| SACOL0139 | capD | 0,43 | capsular polysaccharide synthesis enzyme CapD | 011_0792 | 046_0713 |
| SA0149 | capF | 0,43 | capsular polysaccharide synthesis enzyme CapF | 011_0790 | 046_0715 |
| SAR0158 | cap8H | 0,44 | capsular polysaccharide synthesis enzyme CapH | 011_0788 | 046_0717 |
| SA0145 | capB | 0,45 | capsular polysaccharide synthesis enzyme CapB | 011_0794 | 046_0711 |
| SAR0160 | cap8J | 0,45 | capsular polysaccharide synthesis enzyme CapJ | 011_0786 | 046_0719 |
| SAR0159 | capP | 0,46 | capsular polysaccharide synthesis enzyme CapP | 011_0779 | 046_0726 |
| SA0144 | capA | 0,48 | capsular polysaccharide synthesis enzyme CapA | 011_0795 | 046_0710 |
| SAS0132 | cap8I | 0,48 | capsular polysaccharide synthesis enzyme CapI | 011_0787 | 046_0718 |
| **adhesion genes** |  |  |  |  |  |
| SA2290 | fnbB | 0,28 | FnbB protein |  | 046_2117 |
| SA1003 | fib | 0,31 | Fibrinogen-binding protein | 011_1509 | 046_1264 |
| SA1004 | --- | 0,43 | Fibrinogen-binding protein-related protein | 011_1510 | 046_1263 |
| SA0742 | clfA | 0,45 | Clumping factor A | 011_2325 | 046_2251 |
| SA0519 | sdrC | 2,00 | Ser-Asp rich fibrinogen-binding, bone sialoprotein-binding protein | 011_0417 | 046_1754 |
| SA0520 | sdrD | 4,43 | Ser-Asp rich fibrinogen-binding, bone sialoprotein-binding protein | 011_2683 | 0 |
| **antigen genes** |  |  |  |  |  |
| SA2356 | isaA | 0,41 | immunodominant antigen A | 011_0168 | 046_0166 |
| SA2093 | ssaA2 | 0,46 | Staphylococcal secretory antigen ssaA2 | 011_0580 | 046_1136 |
| SA2097 | --- | 0,39 | Secretory antigen SsaA, putative | 011_0584 | 046_1132 |
| SA2431 | isaB | 2,64 | immunodominant antigen B | 011_1084 | 046_1552 |
| **enzyme genes** |  |  |  |  |  |
| SA2430 | aur | 2,09 | zinc metalloproteinase aureolysin | 011_1083 | 046_1553 |
| SA1726 | --- | 2,37 | Staphostatin A superfamily | 011_1719 | 046_2363 |
| SA1898 | --- | 2,40 | Probable transglycosylase sceD | 011_2359 | 046_1836 |
| SA1725 | scpA | 2,77 | Staphopain A | 011_1718 | 046_2362 |
| SAR0222 | --- | 3,31 | staphylocoagulase precursor | 011_0035 | 046_0518 |
| SA1627 | splF | 8,90 | Serine protease splF | 011_0672 | 046_2496 |
| SAR1902 | splE | 173,71 | Serine protease splE | 011_0673 |  |
| **hemolysin genes** |  |  |  |  |  |
| SA2207 | hlgA | 0,12 | gamma-hemolysin chain II precursor | 011_1955 | 046_0267 |
| SA1007 | hla | 4,26 | alpha-hemolysin | 011_1514 | 046_1259 |
| **toxin genes** |  |  |  |  |  |
| SA0384 | --- | 0,44 | Exotoxin 8 | 011_0060 | 046_0943 |
| SA0383 | set10 | 2,04 | Exotoxin 7 | 011_0061 | 046_0944 |
| **function unknown** |  |  |  |  |  |
| SA2359 | --- | 0,04 | hypothetical protein | 011_0165 | 046_0169 |
| SA2360 | --- | 0,05 | hypothetical protein | 011_0164 | 046_0170 |
| SACOL0348 | --- | 0,08 | hypothetical protein | 011_0484 | 046_2801 |
| SACOL0342 |  | 0,10 | hypothetical protein |  | 046_2795 |
| SA1320 |  | 0,12 | hypothetical protein |  | 046_2732 |
| SA1797 | --- | 0,18 | hypothetical protein | 011_0474 | 046_2771 |
| SA0839 | fabF | 0,19 | hypothetical protein |  | 046_0383 |
| SA1782 | --- | 0,26 | hypothetical protein | 011_1185 | 046_2688 |
| SA2158 | --- | 0,31 | Conserved hypothetical lipoprotein | 011_0656 | 046_1060 |
| SA2280 | --- | 0,44 | Probable exported protein | 011_0241 | 046_2615 |
| SA0395 | --- | 0,45 | hypothetical protein | 011_0735 | 046_1968 |
| SA2281 | --- | 0,47 | Putative membrane protein | 011_0240 | 046_2614 |
| SA0414 | --- | 0,47 | Putative membrane protein | 011_0717 | 046_1950 |
| SA0883 | --- | 0,48 | hypothetical protein | 011_2625 | 046_0425 |
| SAS1871 | --- | 2,00 | Putative membrane protein | 011_1804 |  |
| SA0280 | --- | 2,01 | hypothetical protein | 011_1679 | 046_0655 |
| SA0786 | --- | 2,01 | hypothetical protein | 011_0360 | 046_0455 |
| SA1316 | --- | 2,03 | Putative membrane protein 2 | 011_2321 | 046_1752 |
| SA0524 | --- | 2,07 | putative GTP cyclohydrolase | 011_1606 |  |
| SAOUHSC_02462 | #N/A | 2,08 | hypothetical protein | 011_1832 | 046_1699 |
| SA1162 | --- | 2,10 | hypothetical protein | 011_0983 | 046_0828 |
| SA0543 | --- | 2,10 | Putative membrane protein 1 | 011_1584 | 046_1639 |
| SA1261 | --- | 2,10 | UPF0403 | 011_2603 | 046_1854 |
| SA0199 | --- | 2,11 | hypothetical protein | 011_0743 | 046_0490 |
| SACOL0396 | --- | 2,16 | UPF0189 | 011_1794 | 046_1587 |
| SA1021 | --- | 2,20 | cell division protein MraZ | 011_0824 | 046_1917 |
| SA0749 | --- | 2,21 | hypothetical protein | 011_2073 | 046_2525 |
| SA0315 | --- | 2,21 | hypothetical protein | 011_1793 | 046_1586 |
| SA1514 | --- | 2,21 | hypothetical protein | 011_1289 | 046_0587 |
| SA1178 | --- | 2,23 | UPF0154 | 011_1000 | 046_0845 |
| SA0745 | --- | 2,28 | Putative exported protein | 011_2069 | 046_2529 |
| SAB0259c | --- | 2,31 | hypothetical protein | 011_1643 | 046_0690 |
| SA1153 | --- | 2,43 | hypothetical protein | 011_0968 | 046_0814 |
| SA0275 | --- | 2,52 | Protein essB | 011_1684 | 046_0650 |
| SAV0787 | --- | 2,69 | hypothetical protein | 011_1548 | 046_1001 |
| SA2321 | --- | 2,86 | hypothetical protein | 011_0205 | 046_0128 |
| SA0830 | --- | 2,88 | UPF0344 protein | 011_0313 | 046_0368 |
| SAS056 | --- | 3,01 | Probable exported protein | 011_1734 | 046_2378 |
| SAS025 | --- | 3,03 | Putative membrane protein | 011_0302 | 046_0381 |
| SA1618 | --- | 3,06 | Putative membrane protein | 011_2524 | 046_2547 |
| SA1002 | --- | 3,24 | Hypothetical membrane protein | 011_1508 | 046_1265 |
| SA1345 | --- | 3,36 | UPF0403 | 011_2164 | 046_2150 |
| MW2338 | --- | 3,44 | hypothetical protein | 011_1951 | 046_0263 |
| SA1008 | --- | 3,66 | hypothetical protein | 011_1515 | 046_1258 |
| SA1611 | --- | 4,42 | hypothetical protein | 011_2517 | 046_2554 |
| SA1193 | fmtC | 4,43 | oxacillin resistance-related FmtC protein (pid:87161222) | 011_1017 | 046_0862 |
| SA0748 | hemE | 7,34 | hypothetical protein | 011_2098 | 046_2526 |
| SA1807 | --- | 55,58 | hypothetical protein | 011_0461 |  |
| **mobile genome elements** |  |  |  |  |  |
| **Pathogenicity island** |  |  |  |  |  |
| SA0379 | --- | 0,41 | transposase | 011_0073 | 046_0956 |
| SA1738 | --- | 2,12 | YolD-like protein | 011_1733 | 046_2377 |
| SA1827 | --- | 2,48 | Pathogenicity island protein Orf12 | 011_0422 | 046_0996 |
| SA1826 | --- | 3,06 | pathogenicity island protein (pid:82750114) | 011_0421 | 046_0995 |
| SA1830 | --- | 3,20 | Pathogenicity island protein | 011_1550 | 046_0999 |
| SAR0374 | --- | 5,22 | pathogenicity island protein (pid:82750111) | 011_0424 | 046_0998 |
| SAV0789 | --- | 6,08 | mobile element-associated protein (pid:82751624) | 011_2685 |  |
| SACOL0892 | --- | 7,11 | pathogenicity island protein | 011_2689 | 046_1002 |
| SAR0372 | --- | 26,51 | Pathogenicity island protein Orf17 | 011_2686 |  |
| SACOL0885 | --- | 200,60 | pathogenicity island protein, integrase | 011_2694 |  |
| **Phage** |  |  |  |  |  |
| SAB1756c | --- | 0,00 | phage-like protein 8 |  | 046_2440 |
| SAB1752c | --- | 0,00 | hypothetical protein |  | 046_2785 |
| SAUSA300_1427 | --- | 0,01 | phiSLT ORF86-like protein |  | 046_2788 |
| SAOUHSC_02086 | --- | 0,01 | PV83 orf 4-like protein-related protein |  | 046_2323 |
| SACOL0338 | --- | 0,01 | Phage protein |  | 046_2790 |
| SAOUHSC_02058 | --- | 0,02 | Conserved hypothetical phage protein |  | 046_2027 |
| SAV0851 | --- | 0,02 | Putative phage regulatory protein 1 |  | 046_0694 |
| SAV0862 | --- | 0,02 | Virulence-related phage protein |  | 046_2789 |
| SAB1760 | --- | 0,02 | integrase |  | 046_2435 |
| SAOUHSC_02069 | --- | 0,03 | phi PV83 orf 20-like protein | 011_0479 | 046_2796 |
| SAB1745c | --- | 0,03 | single strand DNA binding protein |  | 046_2831 |
| SAV0872 | --- | 0,03 | phiSLT ORF66-like protein (pid:87160633) |  | 046_2674 |
| SAV0871 | --- | 0,04 | phage-like protein (pid:82751460) | 011_0482 | 046_2844 |
| SACOL0343 | --- | 0,05 | prophage L54a, replicative DNA helicase, putative |  | 046_2794 |
| SAV0879 | --- | 0,05 | Phage77_ORF072 protein |  | 046_2689 |
| SAV0882 | --- | 0,06 | int gene activator RinB |  | 046_2691 |
| SAOUHSC_02067 | --- | 0,08 | bacteriophage L54a, DnaB-like helicase family protein |  | 046_2793 |
| SACOL0347 | --- | 0,08 | Phi ETA orf 25-like protein | 011_0483 | 046_2802 |
| SA1805 | --- | 0,08 | Transcription regulator |  | 046_2321 |
| SA1804 | --- | 0,09 | transcriptional regulator |  | 046_2320 |
| SAV0876 | --- | 0,09 | phi ETA orf 34-like protein | 011_1184 | 046_2729 |
| SAOUHSC_02077 | --- | 0,10 | phi PV83 orf 12-like protein-related protein | 011_0472 | 046_2773 |
| SA1785 | --- | 0,11 | Phi PVL orf 52-like protein | 011_1183 | 046_2730 |
| SAB1733c | --- | 0,12 | phage-like protein 1 |  | 046_2028 |
| SAV1977 | --- | 0,13 | phi PV83 orf 27-like protein |  | 046_2798 |
| SACOL0358 | --- | 0,13 | phage-like protein (pid:82751451) | 011_1185 | 046_2688 |
| SAOUHSC_02205 | --- | 0,14 | Conserved hypothetical phage protein | 011_2612 | 046_2026 |
| SAB1742c | --- | 0,14 | phage protein Gp18 |  | 046_2795 |
| SAOUHSC_02062 | --- | 0,14 | helix-turn-helix DNA binding protein | 011_0481 | 046_2800 |
| SA1788 | --- | 0,15 | Phi PVL orf 50-like protein | 011_0486 | 046_2799 |
| SAS062 | --- | 0,16 | Integrase regulator RinB | 011_1186 | 046_2024 |
| SAV0861 | --- | 0,17 | phage-like protein (pid:82751469) | 011_0474 | 046_2771 |
| SA1781 | --- | 0,19 | Phage_77ORF071 protein | 011_1187 | 046_2023 |
| SA1786 | --- | 0,19 | Phage conserved open reading frame 51 | 011_0487 | 046_2835 |
| SAOUHSC_02203 | --- | 0,23 | Conserved hypothetical phage protein | 011_2615 | 046_2678 |
| SAS1895 | --- | 0,26 | Hypothetical phage protein | 011_1189 | 046_2021 |
| SAB1729c | --- | 0,26 | int gene transcriptional activator | 011_2616 | 046_2679 |
| SAS063 | --- | 0,29 | hypothetical protein | 011_1635 | 046_2667 |
| SAOUHSC_02089 | --- | 0,34 | phage family integrase | 011_0459 | 046_2325 |
| SAS1881 | --- | 0,39 | Hypothetical phage protein | 011_1203 | 046_2007 |
| SAOUHSC_01570 | --- | 0,40 | PVL orf 37-like protein | 011_1636 | 046_2668 |
| SAB1757 | --- | 0,43 | transcriptional repressor | 011_1639 | 046_2439 |
| SA0885 | --- | 2,08 | Abortive infection protein | 011_2628 | 046_0428 |
| SAS1883 | --- | 2,11 | Hypothetical phage protein | 011_1201 | 046_2009 |
| SAB1736c | --- | 2,18 | phage-like protein 3 | 011_1627 | 046_2658 |
| SAS1882 | --- | 2,20 | Hypothetical phage protein | 011_1202 | 046_2008 |
| SAR1545 | --- | 2,51 | Hypothetical phage protein | 011_1634 | 046_2666 |
| SACOL0385 | --- | 2,73 | PhiSLT ORF129-like protein | 011_1807 | 046_1600 |
| MW1402 | --- | 3,03 | terminase small subunit | 011_1828 | 046_1620 |
| SACOL0376 | --- | 3,10 | prophage L54a, major tail protein, putative | 011_1816 | 046_1609 |
| SACOL0375 | --- | 3,38 | prophage L54a, major tail protein, putative 1 | 011_1818 | 046_1611 |
| SAR2094 | --- | 4,17 | Hypothetical phage protein | 011_1638 |  |
| SACOL0889 | --- | 5,23 | phiSLT ORF153-like protein (pid:87161835) | 011_0464 | 046_0693 |
| SAR1543 | --- | 5,70 | Hypothetical phage protein | 011_1632 | 046_2664 |
| SAUSA300_1436 | --- | 5,73 | phiSLT ORF144-like protein, putative lipoprotein | 011_1641 | 046_0692 |
| SAOUHSC_02206 | --- | 9,40 | Hypothetical phage protein | 011_2611 | 046_1902 |

^1^: Genes are classified in GO functional classes and coding sequence number corresponding to the closer available sequenced strain is indicated

^2^: *S. aureus* gene names

^3^: Expression ratio between *S. aureus* O11 and *S. aureus* O46 during log phase (Ratio higher than 2 indicate overexpression in O11 and lower than 0.5 indicate overexpression in O46)

^4^: Names are given according to annotation of available *S. aureus* sequence genomes.

^5^: Coding sequence numbers corresponding to the identified proteins in *S. aureus* O11 and *S. aureus* O46
