# Supplementary material for: Molecular Basis of Virulence in Staphylococcus aureus Mastitis
Source: PLoS One. 2011 Nov 11;6(11):e27354. doi: 10.1371/journal.pone.0027354 (PMC3214034; doi:10.1371/journal.pone.0027354)
Supplement: Table S6 — S. aureus O11 and S. aureus O46 extracellular proteins identified in this study. (DOCX) [file pone.0027354.s006.docx]

**Table S6:** ***S. aureus*** **O11 and *S. aureus* O46** extracellular **proteins identified in this study.**

| Spot^1^ | Description^2^ | O11^3^ | O46^3^ | Loc^4^ | O11 CDS^5^ | O46 CDS^5^ | ED133 ^5^ | PI^6^ | Mass^7^ | Score^8^ | cov.^9^ | #pep.^10^ | EmPAI^11^ |
| --- | --- | --- | --- | --- | --- | --- | --- | --- | --- | --- | --- | --- | --- |
| S1 | Iron-regulated surface determinant protein H | + |  | PSE | 011_1248 | 046_0960 | SAOV_1717 | 5,05 | 100650 | 2209,72 | 42,11 | 37 | 2,58 |
| S2 | Formate acetyltransferase | + |  | C | 011_0041 | 046_0511 | SAOV_0163 | 5,31 | 84808 | 2099,77 | 56,21 | 37 | 5,36 |
| S3 | SspB cysteine protease precursor | + |  | S | 011_2154 | 046_0327 | SAOV_0993c | 5,45 | 42714 | 2027,86 | 65,52 | 30 | 11,52 |
| S4 | AhpC alkyl hydroperoxide reductase subunit C | + |  | C | 011_0085 | 046_0968 | SAOV_0404c | 4,88 | 20963 | 245,60 | 27,51 | 3 | 0,56 |
| S4 | ldh L-lactate dehydrogenase | + |  | C | 011_0136 | 046_0198 | SAOV_2646c | 4,80 | 34399 | 961,14 | 53,61 | 12 | 2,99 |
| S4 | SspB cysteine protease precursor | + |  | S | 011_2154 | 046_0327 | SAOV_0993c | 5,67 | 44548 | 226,11 | 11,96 | 4 | 0,33 |
| S4 | Gap glyceraldehyde-3-phosphate dehydrogenase | + |  | C | 011_2340 | 046_2237 | SAOV_0814 | 4,89 | 36258 | 1006,79 | 50,00 | 13 | 3,06 |
| S5 | SspA glutamyl endopeptidase serine protease | + |  | C | 011_2155 | 046_0325 | SAOV_0994c | 4,68 | 32250 | 1575,55 | 69,26 | 24 | 24,18 |
| S6 | AdhA alcohol dehydrogenase | + |  | C | 011_1554 | 046_0086 | SAOV_0640 | 5,46 | 36024 | 1262,23 | 62,80 | 16 | 3,89 |
| S6 | SspB cysteine protease precursor | + |  | S | 011_2154 | 046_0327 | SAOV_0993c | 5,68 | 44567 | 1169,23 | 47,33 | 17 | 3,18 |
| S6 | Lipoate-protein ligase A | + |  | C | 011_2626 | 046_0426 | SAOV_0973c | 5,19 | 37846 | 65,13 | 6,71 | 1 | 0,09 |
| S7 | Leukocidin F subunit |  | + | S | 011_1752 | 046_1972 | SAOV_2041 | 8,29 | 38639 | 1154,65 | 45,27 | 19 | 11,64 |
| S8 | LukE leukotoxin E subunit | + |  | S | 011_0686 | 046_2483 | SAOV_1813c | 9,38 | 34126 | 483,47 | 16,01 | 7 | 0,91 |
| S8 | Leukocidin chain LukM precursor | + |  | S | 011_1215 | 046_2777 | SAOV_1909 | 9,41 | 35054 | 2215,90 | 69,48 | 32 | 20,47 |
| S8 | Panton-Valentine leukocidin LukF-PV chain precursor | + |  | S | 011_1216 | 046_2776 | SAOV_1908 | 9,16 | 36496 | 1357,08 | 68,01 | 22 | 8,53 |
| S8 | Hla alpha-hemolysin precursor | + |  | S | 011_1514 | 046_1259 | SAOV_1161c | 8,87 | 36329 | 709,86 | 42,24 | 10 | 1,60 |
| S8 | HlgC gamma-hemolysin component C | + |  | S | 011_1956 | 046_0268 | SAOV_2469 | 9,29 | 35562 | 1326,57 | 54,29 | 19 | 4,41 |
| S9 | L-lactate dehydrogenase | + |  | C | 011_0021 | 046_0531 | SAOV_0178 | 5,00 | 34548 | 294,15 | 22,08 | 6 | 0,73 |
| S9 | Fda fructose-1,6-bisphosphate aldolase | + |  | C | 011_0131 | 046_0202 | SAOV_2650 | 5,06 | 32878 | 1446,95 | 64,86 | 18 | 9,08 |
| S9 | Fructose-bisphosphate aldolase | + |  | C | 011_2041 | 046_0921 | SAOV_2166c | 5,01 | 30817 | 533,97 | 37,76 | 9 | 1,79 |
| S10 | 2,3-bisphosphoglycerate-dependent phosphoglycerate mutase |  | + | C | 011_1952 | 046_0264 | SAOV_2463c | 5,23 | 26663 | 545,78 | 36,40 | 7 | 1,28 |
| S10 | YfiA ribosomal subunit interface protein |  | + | C | 011_2483 | 046_1255 | SAOV_0789 | 5,29 | 22143 | 132,31 | 14,66 | 2 | 0,33 |
| S11 | IsaA immunodominant antigen A |  | + | S | 011_0168 | 046_0166 | SAOV_2614c | 5,91 | 24219 | 119,63 | 15,02 | 2 | 0,40 |
| S12 | Gap glyceraldehyde-3-phosphate dehydrogenase | + |  | C | 011_2340 | 046_2237 | SAOV_0814 | 4,89 | 36258 | 95,07 | 6,25 | 2 | 0,42 |
| S13 | CspA cold shock protein | + |  | C | 011_1612 | 046_1035 | SAOV_0833 | 4,51 | 7317 | 281,17 | 78,79 | 3 | 2,32 |
| S14 | IsdC iron-regulated cell surface protein | + |  | PSE | 011_1479 | 046_1293 | SAOV_1127 | 8,94 | 24840 | 245,36 | 21,15 | 4 | 0,88 |
| S14 | SA0570 hypothetical protein | + |  | S | 011_2290 | 046_0078 | SAOV_0649 | 9,17 | 18582 | 56,78 | 6,55 | 1 | 0,18 |
| S15 | Leukocidin S subunit | + |  | S | 011_1753 | 046_1973 | SAOV_2042 | 9,38 | 40379 | 505,70 | 34,19 | 10 | 1,19 |
| S15 | Nuc staphylococcal thermonuclease precursor | + |  | PSE | 011_2070 | 046_2528 | SAOV_0832 | 9,20 | 25089 | 500,44 | 38,16 | 9 | 2,93 |
| S16 | SA0914 hypothetical protein | + |  | S | 011_2000 | 046_0311 | SAOV_1008c | 6,55 | 11338 | 522,48 | 57,14 | 8,00 | 13,10 |
| S17 | N-acetylmuramoyl-L-alanine amidase |  | + | S | 011_1090 | 046_1546 | SAOV_2693 | 5,87 | 69226 | 3098 | 72 | 43 | 17 |
| S18 | IsdA iron-regulated cell wall-anchored protein | + |  | PSE | 011_1476 | 046_1296 | SAOV_1125c | 9,06 | 72162 | 76,28 | 2,17 | 1 | 0,05 |
| S18 | Transmembrane sulfatase | + |  | PSE | 011_2571 | 046_2626 | SAOV_0753 | 9,04 | 74353 | 1666,79 | 39,63 | 24 | 3,54 |
| S19 | Hypothetical protein |  | + | S | 011_0490 | 046_2740 |  | 8,88 | 30854 | 2343,27 | 74,29 | 41 | 65,07 |
| S20 | Fhs formate--tetrahydrofolate ligase |  | + | C | 011_1247 | 046_1352 | SAOV_1718 | 5,76 | 59791 | 708,08 | 23,78 | 11 | 1,01 |
| S20 | PurH bifunctional phosphoribosylaminoimidazolecarboxamide formyltransferase/IMP cyclohydrolase |  | + | C | 011_2552 | 046_2125 | SAOV_1018 | 5,65 | 54284 | 529,95 | 24,39 | 9 | 0,70 |
| S21 | IsdB cell surface transferrin-binding protein | + |  | PSE | 011_1477 | 046_1295 | SAOV_1126c | 9,54 | 39197 | 211,74 | 14,97 | 4 | 0,38 |
| S22 | Leukocidin chain lukM precursor | + |  | S | 011_1215 | 046_2777 | SAOV_1909 | 9,41 | 35054 | 1696,77 | 67,53 | 23 | 8,53 |
| S22 | Sbi IgG-binding protein SBI | + |  | S | 011_1954 | 046_0266 | SAOV_2466 | 9,38 | 49998 | 1001,17 | 36,01 | 16 | 1,77 |

^1^: Spot number (see figure 4)

^2^: Proteins are classified in GO functional classes. Names are given according to annotation of available *S. aureus* sequence genomes.

^3^: +, overexpression of the encoding gene observed in O11 or O46

^4^: predicted protein localization (SurfG+). C, cytoplasmic; S, secreted; PSE, predicted surface exposed

^5^: Coding sequence numbers corresponding to the identified proteins in *S. aureus* O11, *S. aureus* O46, and ED133, respectively.

^6^: Theoretical isoelectric point as determined from the predicted protein sequence

^7^: Theoritical Mass as determined from the predicted protein sequence

^8^: Mascot standard score

^9^: % of the protein sequence covered by the peptides identified

^10^: number of peptides identified

^11^: exponentially modified protein abundance index
