# Supplementary material for: Molecular Basis of Virulence in Staphylococcus aureus Mastitis
Source: PLoS One. 2011 Nov 11;6(11):e27354. doi: 10.1371/journal.pone.0027354 (PMC3214034; doi:10.1371/journal.pone.0027354)
Supplement: Table S7 — Proteins identified by nanoLC MS/MS as being differentially produced by O11 or O46 after analysis of 2D gels of total cell lysate samples (figure S2). (DOC) [file pone.0027354.s007.doc]

**Table S5**: Proteins identified by nanoLC MS/MS as being differentially produced by O11 or O46 after analysis of 2D gels of total cell lysate samples (figure S2)

| 1 | **Description**^2^ | O11^3^ | O46^3^ | **O11 CDS**^4^ | **O46 CDS**^4^ | **ED133 CDS** ^4^ | **PI**^5^ | **Mass**^6^ | **Score**^7^ | **Cov.**^8^ | **#pep.**^9^ | **EmPAI**^10^ |
| --- | --- | --- | --- | --- | --- | --- | --- | --- | --- | --- | --- | --- |
| T1 | clpL ATP-dependent proteinase chain | + |  | 011_0190 | 046_0143 | SAOV_2592 | 4,93 | 75990 | 2579,41 | 62,77 | 33 | 3,40 |
| T1 | transketolase | + |  | 011_0999 | 046_0844 | SAOV_1355 | 4,91 | 72235 | 986,11 | 32,93 | 15 | 0,95 |
| T2 | fabF 3-oxoacyl-ACP synthase II | + |  | 011_0300 | 046_0383 | SAOV_0930 | 5,03 | 43726 | 238,54 | 12,80 | 4 | 0,34 |
| T2 | tuf elongation factor Tu | + |  | 011_0402 | 046_1768 | SAOV_0583 | 4,71 | 43065 | 192,37 | 11,17 | 3 | 0,25 |
| T2 | gatB aspartyl/glutamyl-tRNA amidotransferase subunit B | + |  | 011_0433 | 046_2350 | SAOV_1999 | 5,04 | 53623 | 177,05 | 8,42 | 3 | 0,20 |
| T2 | serS seryl-tRNA synthetase | + |  | 011_1174 | 046_1463 | SAOV_0009 | 5,06 | 48620 | 1226,16 | 48,60 | 18 | 2,48 |
| T2 | UDP-N-acetylmuramoylalanyl-D-glutamyl-2,6- diaminopimelate-D-alanyl-D-alanine ligase | + |  | 011_2374 | 046_1821 | SAOV_2123c | 5,00 | 49899 | 191,39 | 8,85 | 3 | 0,21 |
| T2 | purD phosphoribosylamine--glycine ligase | + |  | 011_2551 | 046_2126 | SAOV_1019 | 4,97 | 45804 | 213,55 | 11,57 | 4 | 0,32 |
| T3 | flavohemoprotein |  | + | 011_0023 | 046_0529 | SAOV_0177 | 5,22 | 42804 | 635,92 | 36,48 | 10 | 1,11 |
| T3 | kbl 2-amino-3-ketobutyrate coenzyme A ligase |  | + | 011_0405 | 046_1766 | SAOV_0585 | 5,14 | 42865 | 85,41 | 3,54 | 1 | 0,08 |
| T3 | pgk phosphoglycerate kinase |  | + | 011_2339 | 046_0078 | SAOV_0815 | 5,17 | 42575 | 1456,44 | 61,87 | 23 | 6,00 |
| T4 | ldh L-lactate dehydrogenase | + |  | 011_0136 | 046_0198 | SAOV_2646c | 4,80 | 34399 | 164,40 | 10,03 | 3 | 0,32 |
| T4 | fabH 3-oxoacyl-acyl carrier protein) synthase III | + |  | 011_0301 | 046_0382 | SAOV_0929 | 4,86 | 33858 | 153,34 | 12,78 | 2 | 0,21 |
| T4 | pgk phosphoglycerate kinase | + |  | 011_2339 | 046_2238 | SAOV_0815 | 4,98 | 42694 | 64,82 | 4,04 | 1 | 0,08 |
| T4 | gap glyceraldehyde-3-phosphate dehydrogenase | + |  | 011_2340 | 046_0591 | SAOV_0814 | 4,89 | 36258 | 164,74 | 8,93 | 2 | 0,19 |
| T5 | fda fructose-1,6-bisphosphate aldolase | + |  | 011_0131 | 046_0202 | SAOV_2650 | 5,06 | 32878 | 1645,30 | 73,31 | 22 | 16,96 |
| T5 | fructose-bisphosphate aldolase | + |  | 011_2041 | 046_0921 | SAOV_2166c | 5,01 | 30817 | 402,09 | 26,57 | 7 | 1,05 |
| T6 | fda fructose-1,6-bisphosphate aldolase | + |  | 011_0131 | 046_0202 | SAOV_2650 | 5,06 | 32878 | 2103,19 | 83,11 | 28 | 46,02 |
| T6 | fructose-bisphosphate aldolase | + |  | 011_2041 | 046_0921 | SAOV_2166c | 5,01 | 30817 | 415,33 | 40,21 | 7 | 1,05 |
| T7 | fda fructose-1,6-bisphosphate aldolase |  | + | 011_0131 | 046_0202 | SAOV_2650 | 4,88 | 33021 | 100,68 | 10,14 | 2 | 0,21 |
| T7 | 2,3-bisphosphoglycerate-dependent phosphoglycerate mutase |  | + | 011_1952 | 046_0264 | SAOV_2463c | 5,23 | 26663 | 1135,53 | 63,16 | 13 | 4,87 |
| T8 | ahpC alkyl hydroperoxide reductase subunit C | + |  | 011_0085 | 046_0968 | SAOV_0404c | 4,88 | 20963 | 608,79 | 53,97 | 8 | 2,83 |
| T8 | frr ribosome recycling factor | + |  | 011_0911 | 046_0757 | SAOV_1261 | 5,04 | 20341 | 253,08 | 20,65 | 4 | 0,85 |
| T8 | sodA superoxide dismutase | + |  | 011_2203 | 046_2189 | SAOV_1553 | 5,08 | 22697 | 134,32 | 17,09 | 2 | 0,32 |
| T9 | ahpC alkyl hydroperoxide reductase subunit C | + |  | 011_0085 | 046_0968 | SAOV_0404c | 4,88 | 20963 | 813,90 | 65,08 | 12 | 5,96 |
| T10 | ahpC alkyl hydroperoxide reductase subunit C |  | + | 011_0085 | 046_0968 | SAOV_0404c | 4,88 | 20963 | 94,94 | 7,41 | 1 | 0,16 |
| T10 | isdB cell surface transferrin-binding protein |  | + | 011_1477 | 046_1295 | SAOV_1126c | 9,54 | 39197 | 481,37 | 22,88 | 7 | 1,07 |
| T11 | rplA 50S ribosomal protein L1 |  | + | 011_0392 | 046_1778 | SAOV_0573 | 9,00 | 24693 | 141,00 | 17,39 | 3 | 0,46 |
| T12 | fda fructose-1,6-bisphosphate aldolase |  | + | 011_0131 | 046_0202 | SAOV_2650 | 4,96 | 32907 | 1368,11 | 66,55 | 19 | 7,31 |
| T12 | dapA dihydrodipicolinate synthase |  | + | 011_2728 | 046_1043 | SAOV_1404 | 4,89 | 32495 | 239,74 | 17,63 | 4 | 0,48 |
| T13 | fda fructose-1,6-bisphosphate aldolase |  | + | 011_0131 | 046_0202 | SAOV_2650 | 4,96 | 32893 | 1885,15 | 79,73 | 25 | 28,06 |
| T13 | dapA dihydrodipicolinate synthase |  | + | 011_2728 | 046_1043 | SAOV_1404 | 4,88 | 32440 | 58,83 | 3,73 | 1 | 0,10 |
| T14 | 2,3-bisphosphoglycerate-dependent phosphoglycerate mutase | + |  | 011_1952 | 046_0264 | SAOV_2463c | 5,23 | 26663 | 867,55 | 59,65 | 11 | 3,64 |
| T15 | glyA serine hydroxymethyltransferase |  | + | 011_2054 | 046_0908 | SAOV_2154c | 5,84 | 45072 | 650,87 | 33,01 | 10 | 1,03 |
| T16 | general stress protein 20U | + |  | 011_1052 | 046_0576 | SAOV_2179c | 4,57 | 16681 | 529,33 | 63,95 | 6 | 3,40 |

^1^: Spot number (see figure S2)

^2^: Protein names are given according to annotation of available *S. aureus* sequence genomes.

^3^: overexpression in *S. aureus* O11 or *S. aureus* O46

^4^: Coding sequence numbers corresponding to the identified proteins in *S. aureus* O11, *S. aureus* O46, and *S. aureus* ED133, respectively.

^5^: Theoretical isoelectric point as determined from the predicted protein sequence

^6^: Theoritical Mass as determined from the predicted protein sequence

^7^: Mascot standard score

^8^: % of the protein sequence covered by the peptides identified

^9^: number of peptides identified

^10^: exponentially modified protein abundance index
