# Supplementary material for: Molecular Basis of Virulence in Staphylococcus aureus Mastitis
Source: PLoS One. 2011 Nov 11;6(11):e27354. doi: 10.1371/journal.pone.0027354 (PMC3214034; doi:10.1371/journal.pone.0027354)
Supplement: Table S8 — Proteins identified by nanoLC MS/MS as being differentially produced by O11 and O46 after analysis of 2D gels of cell wall extract (figure S2). (DOC) [file pone.0027354.s008.doc]

**Table S6**: Proteins identified by nanoLC MS/MS as being differentially produced by O11 and O46 after analysis of 2D gels of cell wall extract (figure S3)

| 1 | **Description**^2^ | O11^3^ | O46^3^ | **O11 CDS**^4^ | **O46 CDS**^4^ | **ED133 CDS** ^4^ | **PI**^5^ | **Mass**^6^ | **Score**^7^ | **Cov.**^8^ | **#pep.**^9^ | **EmPAI**^10^ |
| --- | --- | --- | --- | --- | --- | --- | --- | --- | --- | --- | --- | --- |
| P1 | glyA serine hydroxymethyltransferase | + |  | 011_2054 | 046_0908 | SAOV_2154c | 5,66 | 45091 | 1126,58 | 50,97 | 15 | 1,89 |
| P1 | SA2256 hypothetical protein | + |  | 011_2423 | 046_2101 | SAOV_2518c | 5,47 | 50077 | 58,91 | 3,46 | 1 | 0,07 |
| P2 | mercury(II) reductase | + |  | 011_1575 | 046_1630 | SAOV_0628c | 5,61 | 48318 | 975,72 | 52,50 | 15 | 1,88 |
| P3 | ABC transporter |  | + | 011_0225 | 046_0108 | SAOV_2558 | 5,87 | 25760 | 506,16 | 38,53 | 8 | 2,00 |
| P5 | 2,3-bisphosphoglycerate-dependent phosphoglycerate mutase |  | + | 011_1952 | 046_0264 | SAOV_2463c | 5,23 | 26663 | 953,10 | 59,65 | 11 | 3,64 |
| P6 | asp23 alkaline shock protein 23 |  | + | 011_1851 | 046_1680 | SAOV_2229c | 5,13 | 19180 | 756,71 | 66,27 | 12 | 12,46 |
| P7 | fructose-bisphosphate aldolase |  | + | 011_2041 | 046_0921 | SAOV_2166c | 4,96 | 32893 | 1721,00 | 84,46 |  |  |
| P8 | fructose-bisphosphate aldolase | + |  | 011_2041 | 046_0921 | SAOV_2166c | 4,96 | 32893 | 1345,33 | 72,30 | 25 | 37,78 |
| P9 | aroA bifunctional 3-deoxy-7-phosphoheptulonate synthase/chorismate mutase |  | + | 011_1240 | 046_1346 | SAOV_1723 | 5,83 | 40593 | 564,12 | 31,40 | 8 | 28,06 |
| P10 | Iron-regulated surface determinant protein H | + |  | 011_1248 | 046_0960 | SAOV_1717 | 5,10 | 100639 | 1908,41 | 40,09 | 34 | 2,49 |

^1^: Spot number (see figure S3)

^2^: Protein names are given according to annotation of available *S. aureus* sequence genomes.

^3^: overexpression in O11 or O46

^4^: Coding sequence numbers corresponding to the identified proteins in *S. aureus* O11, *S. aureus* O46, and ED133, respectively.

^5^: Theoretical isoelectric point as determined from the predicted protein sequence

^6^: Theoritical Mass as determined from the predicted protein sequence

^7^: Mascot standard score

^8^: % of the protein sequence covered by the peptides identified

^9^: number of peptides identified

^101^: exponentially modified protein abundance index
