# Supplementary material for: Molecular Basis of Virulence in Staphylococcus aureus Mastitis
Source: PLoS One. 2011 Nov 11;6(11):e27354. doi: 10.1371/journal.pone.0027354 (PMC3214034; doi:10.1371/journal.pone.0027354)
Supplement: Table S9 — Oligonucleotides used in this study for quantitative real-time PCR. (DOC) [file pone.0027354.s009.doc]

**Table S1**: Oligonucleotides used in this study for quantitative real-time PCR

| FtsZ-MW1069-for | CTTCAAATTCATCAAATGCACAA |
| --- | --- |
| FtsZ-MW1069-rev | CGTCTTGTTCTTCTTGAACGTCT |
| clfA-MW0764-for | TTACGAATCAGTTGACGAATGTG |
| clfA-MW0764-rev | AGGCACTGAAAAACCATAATTCA |
| ureA-for | TTACACAACGAGAGCAAGACAAA |
| ureA-rev | TGATTAAAGCTAATGCCTCAGGA |
| phoP-MW1637-for | ATGTCGCAAAAAGTGTTGGTAGT |
| phoP-MW1637-rev | CAAATGCGACAACAACTTCATAA |
| SodA- MW1505-for | TTCTGGGAGTTACTTTCACCAAA |
| SodA- MW1505-rev | CTGCTTTGTCAGCAAATTCTTTT |
| AhpF for | CGAAGAATTTGGCAATGGTC |
| AhpF rev | ACCACTAGCAGGACCACCAC |
| SigS For | AAACCCTCAAGCAAGCAATC |
| SigS rev | TGGTTGTTCAGTTAATGTTGGTG |
| GyrB For | TATGGTGCTGGGCAAATACA |
| GyrB Rev | TATGGTGCTGGGCAAATACA |
| SirA For | TGCATTCCAAAAAGATGCAA |
| SirA Rev | TTTCACCAGCATATCCACCA |
| AgrA For | CCTCGCAACTGATAATCCTTATG |
| AgrA Rev | ACGAATTTCACTGCCTAATTTGA |
| cap8A For | GCGCTATTGTTACATTTTTCGTC |
| cap8A Rev | TCTTGTGCCATAAACTGAGGATT |
| hld | TAAGGAAGGAGTGATTTCAATGG |
| hld | GTGAATTTGTTCACTGTGTCGAT |
| hu | AGAAGCTGGTTCAGCAGTAGATG |
| hu | TACCTCAAAGTTACCGAAACCAA |
| gyra | ACATGCATCATTAATTCGATTCC |
| gyra | TACATCAAGCCCTACAACTTCGT |
