# Supplementary figures and images for: Molecular Basis of Virulence in Staphylococcus aureus Mastitis
Source: PLoS One. 2011 Nov 11;6(11):e27354. doi: 10.1371/journal.pone.0027354 (PMC3214034; doi:10.1371/journal.pone.0027354)

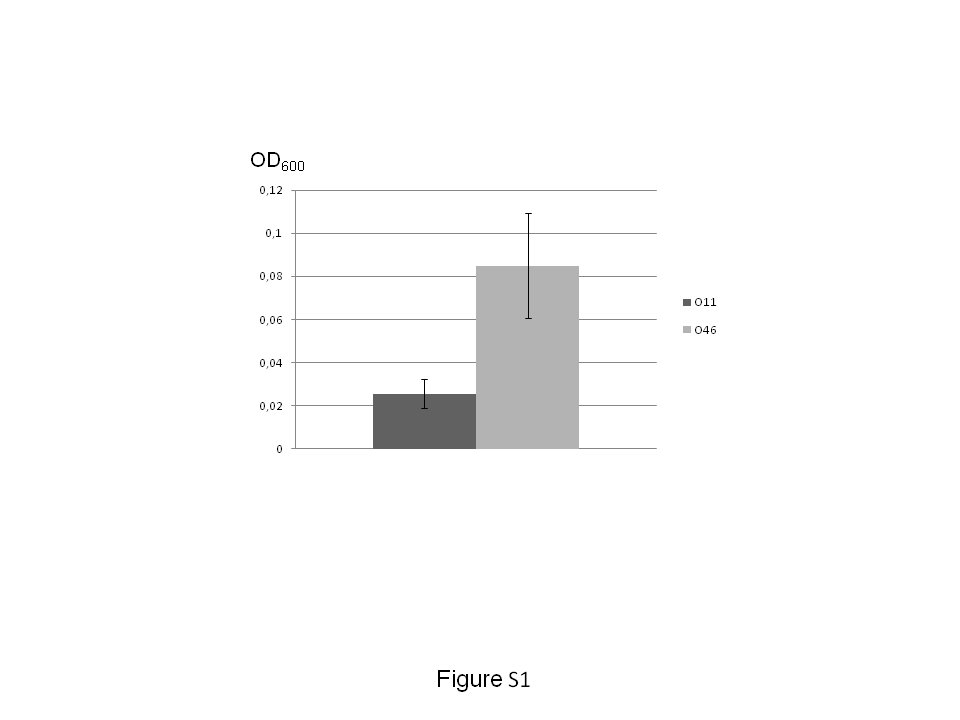

Supplement: Figure S1 — Biofilm production in S. aureus O11 and O46 as determined by Cristal violet staining assay. Biofilm staining assays were performed as described previously [29]. Briefly, after bacterial growth in iron-depleted RPMI, microtiter plates (MultiwellTM 6 well, Becton Dickinson) were washed twice with phosphate-buffered saline (PBS), fixed for 20 min at 80°C and stained for 10 min with 1% (w/v) crystal violet solution freshly diluted twofold in 1% (v/v) ethanol/distilled water. Plates were then washed with water and photographed. The crystal violet was dissolved in dimethyl sulfoxide (DMSO) for 1 h before OD600 nm measurements. Biofilm formation was estimated for each strain, on 6 replicates, and the data were analysed by the student's paired t test. A P value of 0.05 or less (here, P = 0.044).was considered statistically significant. (TIF) [file pone.0027354.s010.tif]

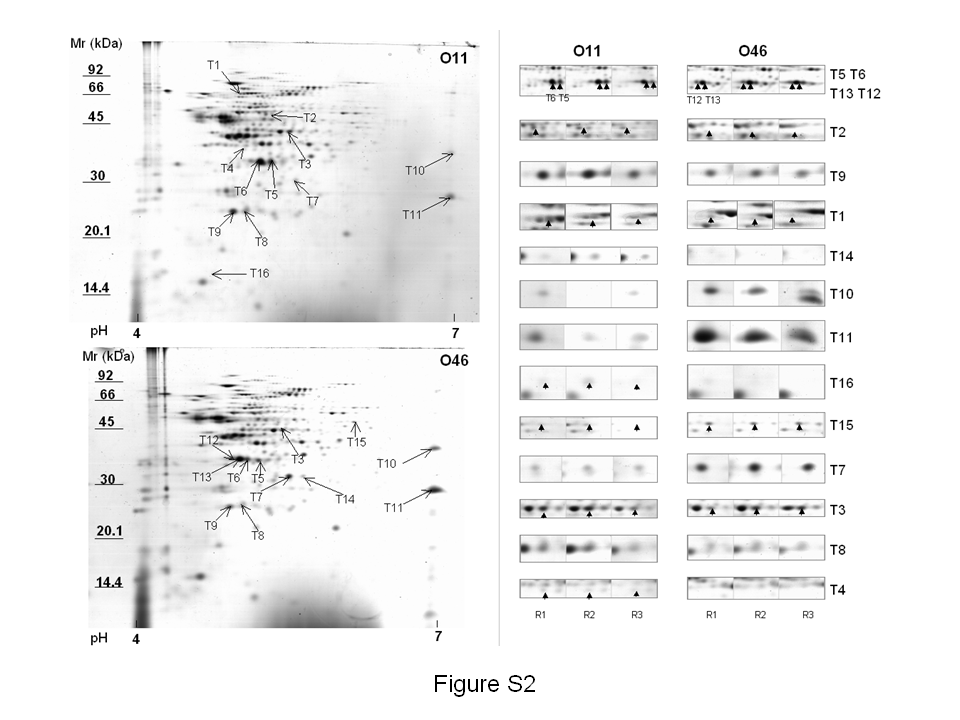

Supplement: Figure S2 — Proteomic comparison of S. aureus O11 and S. aureus O46 cell lysates. A: representative 2-DE gel of S. aureus O11 (upper gel) and S. aureus O46 (lower gel) total lysates. Proteins were prepared after growth in iron-depleted RMPI. 200 µg of protein preparation was separated on 13 cm gels (pI 4–7, 14% SDS-PAGE) and Coomassie Blue-stained. Image analysis with Image Master 2D revealed differences in the protein spots indicated with arrows and numbers. Identification was carried out by NanoLC MS/MS (see Table S7). B: The expression of numbered spots are depicted in three different gels prepared from three biological replicates (R1, R2, R3) from O11 or O46 total lysates. (TIF) [file pone.0027354.s011.tif]

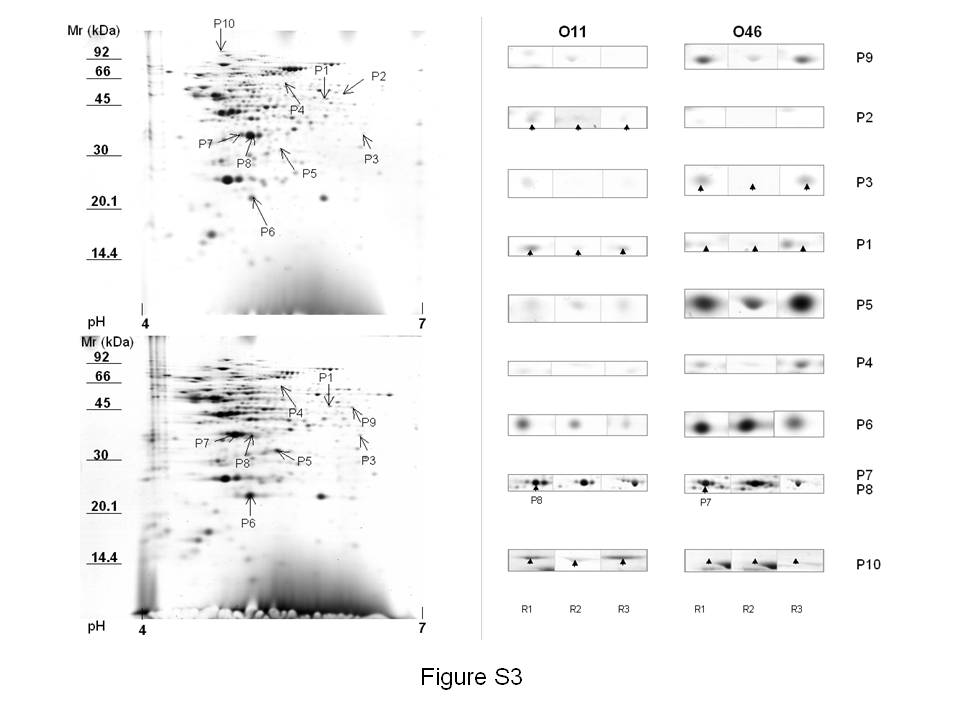

Supplement: Figure S3 — Proteomic comparison of S. aureus O11 and S. aureus O46 cell wall proteins. A: representative 2D gel of O11 (upper gel) and O46 (lower gel) cell wall extracts. Proteins were prepared after growth in iron-depleted RMPI. 200 µg of protein preparation was separated on 13 cm gels (pI 4–7, 14% SDS-PAGE) and Coomassie Blue-stained. Image analysis with Image Master 2D revealed differences in the protein spots indicated with arrows and numbers. Identification was carried out by NanoLC MS/MS (see Table S8). B: The expression of numbered spots are depicted in three different gels prepared from three biological replicates (R1, R2, R3) from O11 or O46 cell wall samples. (TIF) [file pone.0027354.s012.tif]

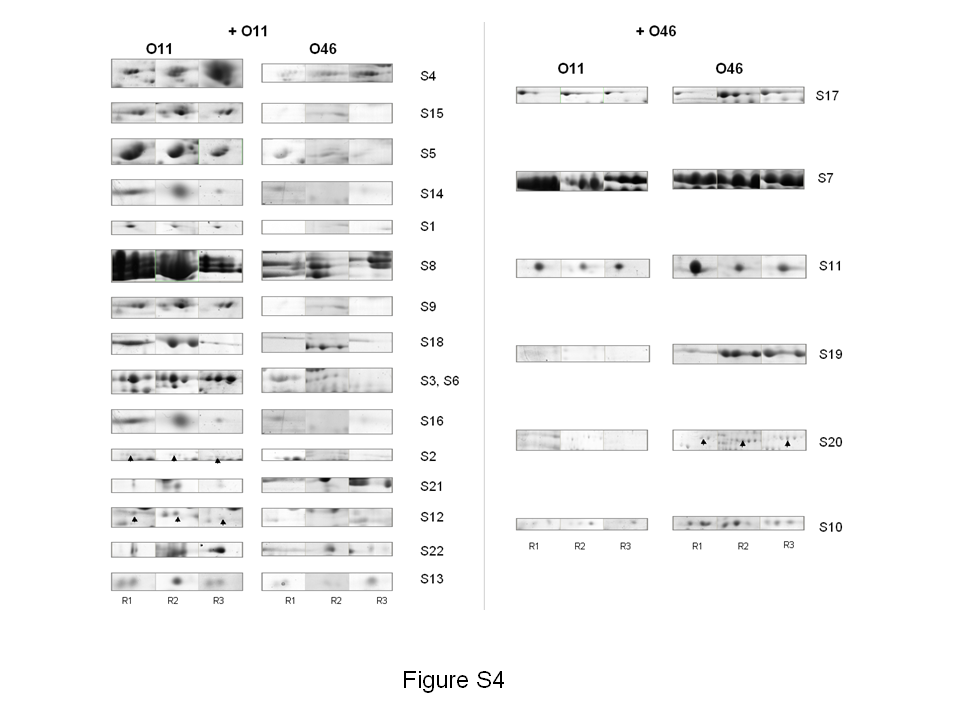

Supplement: Figure S4 — Proteomic differences between S. aureus O11 and S. aureus O46 extracellular proteins highlighted by image analysis with Image master 2D. Identification was carried out by NanoLC MS/MS (see Table S6). The expression of numbered spots are depicted in three different gels prepared from three biological replicates (R1, R2, R3) from O11 (left panel) or O46 (right panel) extracellular protein samples. (TIF) [file pone.0027354.s013.tif]

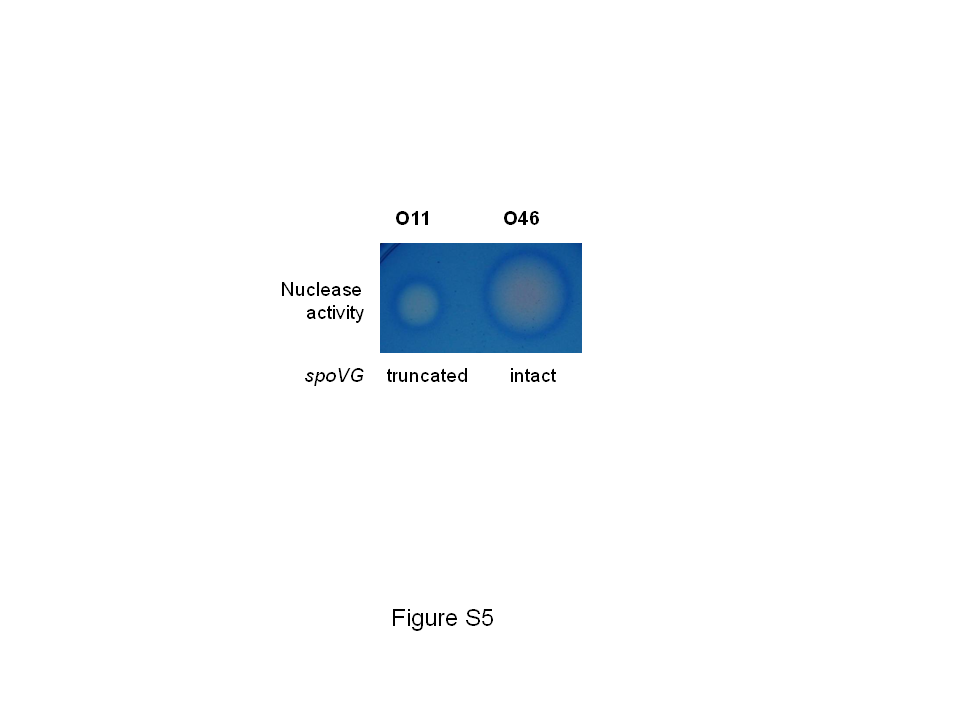

Supplement: Figure S5 — Nuclease activity assay on supernatant of O11 and O46. A nuclease plate assay was carried out on supernatant of O11 and O46 strains after overnight culture on deferoxamine-RPMI. 10 µL of 0.2 µm filtered supernatant were spotted on Toluidine Blue-DNA agar as described previously [72]. Plates were incubated o.n. at 37°C and nuclease activity was revealed by the development of a pink halo, which diameter is proportional to the amount of Nuclease secreted. Presence of truncated spoVG in O11 correlates with a lower nuclease production as previously reported [46]. (TIF) [file pone.0027354.s014.tif]
